# Whole-Genome Sequencing-Based Antimicrobial Resistance Characterization and Phylogenomic Investigation of 19 Multidrug-Resistant and Extended-Spectrum Beta-Lactamase-Positive *Escherichia coli* Strains Collected From Hospital Patients in Benin in 2019

**DOI:** 10.3389/fmicb.2021.752883

**Published:** 2021-12-09

**Authors:** Carine Laurence Yehouenou, Bert Bogaerts, Sigrid C. J. De Keersmaecker, Nancy H. C. Roosens, Kathleen Marchal, Edmond Tchiakpe, Dissou Affolabi, Anne Simon, Francis Moise Dossou, Kevin Vanneste, Olivia Dalleur

**Affiliations:** ^1^Clinical Pharmacy Research Group (CLIP), Louvain Drug Research Institute (LDRI), Université Catholique de Louvain UCLouvain, Brussels, Belgium; ^2^Laboratoire de Référence des Mycobactéries (LRM), Cotonou, Benin; ^3^Faculté des Sciences de la Santé (FSS), Université d’Abomey Calavi (UAC), Cotonou, Benin; ^4^Transversal activities in Applied Genomics, Sciensano, Brussels, Belgium; ^5^Department of Plant Biotechnology and Bioinformatics, Ghent University, Ghent, Belgium; ^6^Department of Information Technology, IDLab, Ghent University, IMEC, Ghent, Belgium; ^7^Department of Genetics, University of Pretoria, Pretoria, South Africa; ^8^Laboratory of Cell Biology and Physiology, Department of Biochemistry and Cellular Biology Faculty of Sciences and Technology and Institute of Applied Biomedical Sciences (ISBA), University of Abomey-Calavi, Cotonou, Benin; ^9^National Reference Laboratory of Health Program Fighting Against AIDS in Benin, Health Ministry, Cotonou, Benin; ^10^Centre National Hospitalier et Universitaire Hubert Koutoukou Maga (CNHU-HKM), Cotonou, Benin; ^11^Centres hospitaliers Jolimont, prevention et contrôle des infections, Haine-Saint-Paul, Belgium; ^12^Department of Surgery and Surgical Specialties, Faculty of Health Sciences, Campus universitaire champ de foire, Cotonou, Benin; ^13^Pharmacy, Clinique universitaire Saint-Luc, Université catholique de Louvain, UCLouvain, Brussels, Belgium

**Keywords:** *Escherichia coli*, whole-genome sequencing, extended-spectrum beta-lactamases, antimicrobial resistance, Benin

## Abstract

The increasing worldwide prevalence of extended-spectrum beta-lactamase (ESBL) producing *Escherichia coli* constitutes a serious threat to global public health. Surgical site infections are associated with high morbidity and mortality rates in developing countries, fueled by the limited availability of effective antibiotics. We used whole-genome sequencing (WGS) to evaluate antimicrobial resistance and the phylogenomic relationships of 19 ESBL-positive *E. coli* isolates collected from surgical site infections in patients across public hospitals in Benin in 2019. Isolates were identified by MALDI-TOF mass spectrometry and phenotypically tested for susceptibility to 16 antibiotics. Core-genome multi-locus sequence typing and single-nucleotide polymorphism-based phylogenomic methods were used to investigate the relatedness between samples. The broader phylogenetic context was characterized through the inclusion of publicly available genome data. Among the 19 isolates, 13 different sequence types (STs) were observed, including ST131 (*n* = 2), ST38 (*n* = 2), ST410 (*n* = 2), ST405 (*n* = 2), ST617 (*n* = 2), and ST1193 (*n* = 2). The *bla*_CTX-M-15_ gene encoding ESBL resistance was found in 15 isolates (78.9%), as well as other genes associated with ESBL, such as *bla*_OXA-1_ (*n* = 14) and *bla*_TEM-1_ (*n* = 9). Additionally, we frequently observed genes encoding resistance against aminoglycosides [*aac-(6')-Ib-cr*, *n* = 14], quinolones (*qnrS_1_*, *n* = 4), tetracyclines [*tet*(*B*), *n* = 14], sulfonamides (*sul2*, *n* = 14), and trimethoprim (*dfrA17*, *n* = 13). Nonsynonymous chromosomal mutations in the housekeeping genes *parC* and *gyrA* associated with resistance to fluoroquinolones were also detected in multiple isolates. Although the phylogenomic investigation did not reveal evidence of hospital-acquired transmissions, we observed two very similar strains collected from patients in different hospitals. By characterizing a set of multidrug-resistant isolates collected from a largely unexplored environment, this study highlights the added value for WGS as an effective early warning system for emerging pathogens and antimicrobial resistance.

## Introduction

Multidrug-resistant (MDR) and extended-spectrum beta-lactamase (ESBL) producing bacteria pose a growing clinical and public health risk (Pitout and Laupland, 2008). In 2017, the World Health Organization (WHO) listed ESBL-producing *Enterobacterales*, including *E. coli*, as pathogens of critical priority for research and development of antibiotics (Asokan et al., 2019). ESBLs confer resistance to penicillins; to first-, second-, third-, and fourth-generation cephalosporins; and to aztreonam (but not to the cephamycins or carbapenems; Brolund, 2014). ESBL genes are commonly observed in association with genes that confer resistance to other classes of antibiotics, resulting in multidrug resistance (Harbarth et al., 2015). Additionally, they are easily transferred between bacteria through horizontal gene transfer (Brolund, 2014).

Two classification systems for beta-lactamases are currently in use. The most widely used is the Ambler molecular classification (Hall and Barlow, 2005), which defines four molecular classes of beta-lactamases based on amino-acid homology (A, B, C, and D). The most prevalent ESBLs belong to class A, which includes the Cefotaximase-Munich (CTX-M), Temoneria (TEM), and Sulfhydryl Variable (SHV) families.

Hospital-acquired infections of ESBL-producing *Enterobacterales* were first described in 1983 (Bradford, 2001), where resistance arose from point mutations in plasmid-mediated genes encoding beta-lactamase enzymes. These enzymes include TEM-1, TEM-2, and SHV-1, as well as CTX-M, which later became predominant worldwide (Hall and Barlow, 2005). Studies have shown that CTX-M-15-producing *E. coli* comprises one of the most prevalent ESBL-producing *Enterobacterales* (Cantón et al., 2012; Harbarth et al., 2015; Nwafia et al., 2019) and that the global dissemination of ESBL-producing *E. coli* is associated with specific clones mainly assigned to sequence types 131 (ST131) and 405 (ST405; Naseer and Sundsfjord, 2011). As these resistant strains are associated with higher mortality rates, extended hospital stays, and higher health costs, the burden of these infections is enormous.

Since only a few molecular typing studies have examined *E. coli* related infections in hospitals in low- and middle-income countries (LMICs), such as Benin, limited information is available on the subtypes that cause infections and their transmission dynamics in LMICs. Genotypic antimicrobial resistance evaluation has been performed mainly by polymerase chain reaction (PCR), which often fails to capture the full complexity of antimicrobial genetic structures (Anago et al., 2015; Koudokpon et al., 2018). In recent years, whole-genome sequencing (WGS) has increasingly been used to examine drug-resistant commensal and pathogenic *E. coli*, providing more complete insight into the genetic structures associated with the evolution of multidrug resistance (MDR) and transmission dynamics (Punina et al., 2015). In Benin, this information is currently lacking, as WGS-based studies are rarely performed. Therefore, we performed WGS on 19 ESBL-positive strains of clinical *E. coli* isolated from surgical site infections from patients in four public hospitals and characterized their phenotypic and genotypic AMR profiles, and phylogenomic diversity.

## Materials and Methods

### Study Overview and Ethics Consent

This study was part of a larger project (Multidisciplinary Strategy for Prevention and Infection Control: MUSTPIC) conducted from April 2018 to January 2020 to explore etiological bacteria involved in surgical site infections in six public hospitals in Benin. Overall, 229 isolates were collected in the context of this project. This study was approved by the Ethics Committee of the Faculty of Health Sciences (FSS, Benin) under reference number: 012-19/UAC/FSS/CER-SS. Written informed consent was obtained from each participant before enrolment into the study.

### Sample Collection and Species Identification

Samples were originally collected as wound swabs between January 2019 and January 2020 from patients that had consented to participate. We included the obstetric (particularly caesarean sections) and gastrointestinal wards at four of these six public hospitals in Benin. To maintain confidentiality, each of these hospitals was randomly assigned a letter A-D. These wards were chosen because caesarean sections are one of the most common surgical procedures, and gastrointestinal wards were present in all the hospitals. All participating hospitals are located in the south of Benin, thereby allowing daily transport from each hospital to the CNHU-HKM laboratory, where all wound swabs were initially collected and analyzed. All identifications were confirmed in Belgium using Matrix-Assisted Laser Desorption Ionization-Time of Flight (MALDI-TOF) mass spectrometry (Brucker daltonics, Bremen, Germany) employing a threshold of ≥ 2.0. Out of 229 samples, 49 were identified as *Staphylococcus aureus* and 180 as belonging to the *Enterobacterales*, of which 62 samples were identified as *E. coli*.

### Antimicrobial Susceptibility Testing

The 62 isolates identified as *E. coli* were tested for susceptibility toward 16 antimicrobials using the modified Kirby-Bauer disk diffusion method according to the EUCAST guidelines (EUCAST, 2015). *Escherichia coli* strain ATCC 25922 was used for quality control. The following antimicrobial disks (Bio-Rad, Marnes-la-coquette, France) were used amikacin (30 μg), amoxicillin-clavulanic acid (20/10 μg), ampicillin (10 μg), cefepime (30 μg), cefotaxime (30 μg), cefoxitin (30 μg), ceftriaxone (30 μg), chloramphenicol (30 μg), ciprofloxacin (5 μg), gentamycin (10 μg), imipenem (10 μg), levofloxacin (5 μg), meropenem (10 μg), piperacillin (100 μg), tobramycin (10 μg), and trimethoprim + sulfamethoxazole (25 μg). Isolates that were resistant to at least three different classes of antimicrobials were considered as MDR (Magiorakos et al., 2012). Antimicrobial classes for 15 of the 16 tested antibiotics were assigned based on the ResFinder classification.^1^ Levofloxacin was missing from the aforementioned classification and manually assigned to fluoroquinolones. The ESBL phenotype was identified by the double-disk synergy method on Mueller Hinton agar using ceftazidime and ceftriaxone placed at 20 mm apart from a disk containing amoxicillin and clavulanic acid. A clear-cut enhancement of the inhibition in front of either ceftazidime and/or ceftriaxone disks toward the clavulanic acid-containing disk (also called “champagne-cork” or “keyhole”) was interpreted as positive for ESBL production. Additionally, isolates were tested for the presence of the beta-lactamase *ampC* phenotype using the cefoxitin-cloxacillin disk diffusion test, as described previously (Thean et al., 2009). Out of the 62 *E. coli* isolates, 43 exhibited ESBL-positive phenotypes. A subset of 19 isolates was then selected for WGS analysis based on their phenotypic resistance out of these ESBL-positive phenotypes in order to increase the chance of inferring transmission dynamics for this subset of strains, while also trying to retain samples highly resistant to different antibiotics and originating from the four different hospitals. An overview of the selected isolates is provided in Table 1.

**Table 1:** Overview of the 19 samples sequenced in this study.

Sample name	Hospital	Ward	Collection date	Surgical intervention	Patient age	Patient sex
s_12116	D	GI	October 15, 2019	Peritonitis	18	Female
s_12117	D	GI	October 15, 2019	Peritonitis	18	Male
s_12155	C	M	October 16, 2019	Caesarean	26	Female
s_12301	C	M	October 20, 2019	Caesarean	35	Female
s_12414	B	GI	October 22, 2019	NA	36	Male
s_12479	C	M	October 23, 2019	Caesarean	22	Female
s_12480	D	M	October 23, 2019	Caesarean	34	Female
s_12845	D	GI	October 31, 2019	Appendicitis	18	Male
s_12849	D	GI	October 31, 2019	Appendicitis	21	Male
s_13022	D	GI	November 08, 2019	Evisceration	55	Female
s_13150	A	GI	November 08, 2019	Peritonitis	40	Female
s_13959	A	GI	November 26, 2019	Appendicitis	52	Female
s_13987	B	GI	November 26, 2019	Appendicitis	23	Female
s_3117	A	M	November 12, 2019	Caesarean	32	Female
s_316	D	GI	November 28, 2019	Appendicitis	23	Male
s_317	D	GI	June 06, 2019	Appendicitis	33	Male
s_4294	D	GI	June 04, 2019	NA	36	Male
s_6558	D	GI	May 30, 2019	NA	35	Male
s_90	A	M	November 28, 2019	Caesarean	28	Female

*M, maternal; GI, gastro-internal; and NA, Not available*.

### Whole-Genome Sequencing

DNA extraction for selected isolates was done by using the Qiagen universal Biorobot (Limburg, Netherlands) according to the manufacturer’s instructions. Isolate sequencing libraries were created using Nextera XT DNA library preparation (Illumina, San Diego, CA) according to the manufacturer’s instructions, and subsequently underwent Illumina sequencing using the MiSeq V3 chemistry (Illumina, San Diego, CA) for production of 2 × 250 bp paired-end reads. All sequencing data have been submitted to SRA (Leinonen et al., 2011) under BioProject PRJNA701417, and individual accession numbers are provided in Supplementary Table S1.

### WGS-Based Isolate Characterization

All WGS reads were trimmed and *de novo* assembled using SPAdes (Bankevich et al., 2012) as described in Bogaerts et al. (2021). Detection of AMR genes was performed as described in Bogaerts et al. (2019) using the sequences from the NCBI NDARO database (downloaded on March 27, 2020; Feldgarden et al., 2019). Hits with less than 90% sequence identity or less than 90% target coverage were filtered out. A local installation of PointFinder (checked out from BitBucket on February 27, 2019; Zankari et al., 2017) was used to detect mutations associated with AMR. Phenotypic resistance was predicted when at least one gene or mutation was detected with resistance to the corresponding antibiotic(s). Isolates were then typed using the methodology described in Bogaerts et al. (2019) using the classic MLST and cgMLST schemes from EnteroBase (Zhou et al., 2020), downloaded on the September 6, 2020. The putative genetic origin (i.e., chromosomal or plasmidic) of the detected resistance genes was evaluated by classifying the corresponding contigs using the “mob-recon” command from MOB-suite 3.0.0 with default settings (Robertson and Nash, 2018).

The samples were screened for contaminants using Kraken 2 2.0.7 (Wood and Salzberg, 2014) with default parameters and an in-house database containing all NCBI RefSeq Genome entries with the “Complete Genome” assembly level (database accessed February 18, 2019; O’Leary et al., 2016) accession prefixes NC, NW, AC, NG, NT, NS, and NZ of the following taxonomic groups: archaea, bacteria, fungi, human, protozoa, and viruses. Samples for which Kraken 2 classified more than 5% of reads to a species other than *E. coli* were considered contaminated. For these samples, reads classified as the contaminant species (up to the genus level) were removed before the *de novo* assembly. All subsequent analyses were performed on these cleaned assemblies.

### Comparison of Phenotypic and Genotypic Predicted AMR Susceptibility

Correspondence with observed phenotypic resistance was evaluated using the following definitions: true positive (TP) and false negative (FN) as cases with phenotypic resistance to an antibiotic where the WGS-based workflow predicted resistance and susceptibility, respectively, and true negative (TN) and false positive (FP) as cases with phenotypic susceptibility to an antibiotic where the WGS-based workflow predicted susceptibility and resistance, respectively. This comparison was limited to six of the tested antibiotics for which the NDARO database or PointFinder databases contained at least one entry. For the remaining 10 antibiotics, the WGS workflow could only perform predictions at the level of the parent class since the database only contains information up to this level. For example, the NDARO database contains genes annotated as associated with resistance to cephalosporins, but it does not provide information on resistance to specific members of this class, such as cefepime or cefotaxime.

### Phylogenomic Analysis

The relatedness between samples from this study was first determined by constructing phylogenies based on cgMLST results. Allele matrices were filtered by removing samples with less than 90% of loci detected and afterward removing loci detected in less than 90% of samples. Minimum spanning trees (MSTs) were then constructed from the filtered allele matrices using GrapeTree 2.2 (Zhou et al., 2017) with the “method” option set to “MSTreeV2.” The phylogeny was visualized and annotated in the web-based iTOL platform (Letunic and Bork, 2019). The broader phylogenomic context of samples was also investigated by including all assemblies assigned to any of the STs detected in our samples collected after 2018 in Africa or Europe, retrieved from EnteroBase on January 19, 2021. Initially, only samples from Africa were collected, but since only very few samples matched these criteria, the dataset was extended with samples from Europe. Separate MSTs were then constructed for the six STs with at least two samples from this study (ST38, ST131, ST405, ST410, ST617, and ST1193) using the same methodology as described above. Additionally, Single-nucleotide polymorphism (SNP) addresses were determined using SnapperDB 1.0.6 (Ashton et al., 2015) and PHEnix v1.4.1,^2^ as described previously (Nouws et al., 2020), for all newly sequenced samples and genomes retrieved from EnteroBase with available WGS Illumina data. An additional filtering step was introduced to remove SNPs located in regions of the reference genomes flagged as prophages by PHAST (Zhou et al., 2011) with default settings. The SNP address is a strain level 7-digit nomenclature based on the number of pairwise SNP differences. Each digit represents the cluster membership for the given number of SNP differences, starting (right to left) with 0 (i.e., no SNP differences) to 5, 10, 25, 50, 100, and 250. Isolates sharing the same cluster digit differ by fewer than the corresponding number of SNPs (Ashton et al., 2015). An example of the SNP address methodology is provided in Supplementary Figures S2–S3. The minimum required average mapping depth was lowered to 25x to ensure all samples from this study could be analyzed. Reference genomes from the corresponding ST were selected randomly from all genomes available in EnteroBase (Supplementary Table S2) for the respective STs but only considering complete genomes. Sequences with a “plasmid” annotation in the header were removed from the reference genomes before running the SNP typing analysis. AMR prediction for public genomes was performed as described above (phenotypic AMR susceptibility information was not available for these genomes).

## Results

### Phenotypic Antimicrobial Susceptibility Testing

**Figure 1:**
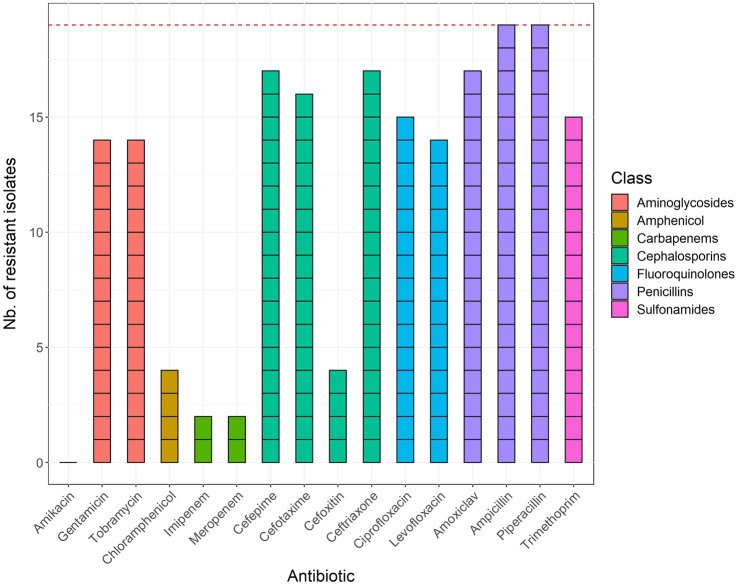
Results of phenotypic antimicrobial susceptibility testing of the 19 sequenced *E. coli* isolates. Bars are colored according to the corresponding class of antibiotics. The red horizontal line corresponds to the total number of isolates (*n* = 19). Trimethoprim + sulfamethoxazole was abbreviated to trimethoprim and amoxicillin + clavulanic acid to amoxiclav.

An overview of AMR susceptibility testing results of the 19 selected ESBL-positive isolates is provided in Figure 1. All 19 isolates exhibited resistance to ampicillin and piperacillin. Most samples also displayed low susceptibility to amoxicillin-clavulanic acid, cefepime, cefotaxime, ceftriaxone, ciprofloxacin, gentamycin, levofloxacin, tobramycin, and trimethoprim + sulfamethoxazole. Four isolates showed resistance to cefoxitin and chloramphenicol, while two isolates showed resistance to imipenem and meropenem. None of the tested isolates exhibited resistance to amikacin.

### Isolate Characterization Based on WGS Data

Overviews of read trimming and *de novo* assembly statistics for the sequenced isolates are provided in Supplementary Tables S3 and S4, respectively. Assembly statistics indicated generally high quality, with a median total assembly length of 5,064,374 bp and N50 of 168,657 bp. Samples s_316 and s_4294 were outliers with a larger total assembly length (9,568,828 bp and 8,564,805 bp, respectively) and smaller N50 (40,329 bp and 69,875 bp, respectively). Kraken 2 analysis indicated that these samples also contained reads from *Acinetobacter* besides *E. coli*, likely explaining their increased cumulative assembly lengths and lower N50. Notwithstanding, only a minor fraction of the total reads was identified as *Acinetobacter*, indicating that enough *E. coli* reads were present in both samples to be retained for further analysis. Reads classified as *Acinetobacter* were therefore removed before performing a new *de novo* assembly, which was used for further analysis for these two samples. We performed additional analysis to confirm that the contamination indicated by Kraken 2 in these two samples was not due to a plasmid, but rather due to the presence of an *Acinetobacter baumannii* strain, for which results are provided in the Supplementary Material. The 19 samples were classified into 13 different STs: ST10 (n: 1), ST38 (n: 2), ST127 (n: 1), ST131 (n: 2), ST167 (n: 1), ST354 (n: 1), ST405 (n: 2), ST410 (n: 2), ST569 (n: 1), ST617 (n: 2), ST648 (n: 1), ST1193 (n: 2), and ST2659 (n: 1), as indicated in Supplementary Table S5.

### Genotypic WGS-Based AMR Characterization

We detected several beta-lactam resistance genes across our samples, including *bla*_CTX-M-15_, *bla*_CMY-42_, *bla*_EC_, *bla*_OXA-1_, *bla*_OXA-181_, and *bla*_TEM-1_. We also detected the *aph(3″)-Ib* (68.4%, n = 13) and *aph(6)-Id* (73.7%, n = 14) genes associated with resistance to streptomycin, in the large majority of samples, as well as the *aac(6′)-Ib-cr* gene (73.7%, n = 14), responsible for a reduction in ciprofloxacin activity. Four different fluoroquinolone resistance determinants were observed [*qnrS1*, *qepA8*, *qepA4*, and *aac*(*6′*)*-Ib-cr*]. Other AMR genes that were detected were associated with trimethoprim resistance (*dfrA8*, *dfrA12*, *dfrA14*, and *dfrA17*), macrolide resistance *mph(A)*, sulphonamide resistance (*sul1*, *sul2*), tetracycline resistance *tet(A)* and *tet(B)*, and phenicol resistance (*catA1*). Additionally, 16 isolates carried at least one nonsynonymous mutation in the housekeeping genes *parC* S80I, *gyrA* S83L, *gyrA* D87N, and *parE* S458A, which are associated with resistance to fluoroquinolones.

A complete overview of the detected AMR genes, point mutations, and predicted phenotypes with WGS is provided in Supplementary Table S6. The majority of resistance genes was located on contigs with a plasmid origin predicted by MOB-suite, as indicated in Supplementary Table S7. Resistance was most commonly predicted for cephalosporins (all 19 samples), tetracycline (*n* = 18), and quinolones (*n* = 17).

### Comparison of Phenotypic and Genotypically Predicted AMR Susceptibility

Full results for comparing genotypically predicted and observed phenotypic AMR profiles are provided in Supplementary Table S8. Out of 62 resistant phenotypes, 60 were predicted correctly (i.e., TP). Sample s_12117 was incorrectly predicted as sensitive to gentamicin, and sample s_4294 to tobramycin. While sample s_4294 was flagged as contaminated by our Kraken 2 analysis, we confirmed that these mismatches were not caused by the additional filtering step because no associated genes were presented in unfiltered assemblies (results not shown). Out of 52 susceptible phenotypes, 34 were predicted correctly (i.e., TN). The large majority of the 18 FP was limited to amikacin (*n* = 14) and caused by the detection of the *aac*(*6′*)*-Ib* gene in susceptible samples. The presence of this gene likely does not suffice for phenotypic resistance at the tested dosage (see also section “Discussion”). Similarly, for resistance to ciprofloxacin, the two FP samples, s_13022 and s_90, only carried the *gyrA* S83L mutation, while TP samples also carried multiple other mutations, including *gyrA* D87N, *parC* S80I, and *parE* L416F. Combining the predictions across all samples resulted in WGS-based prediction accuracy of 82.5%, precision of 76.9%, sensitivity of 96.8%, and specificity of 65.4%. When discarding the results for amikacin, the accuracy increased to 93.6%, precision to 93.8%, sensitivity to 96.8%, and specificity to 87.5%.

### Phylogenomic Analysis

**Figure 2:**
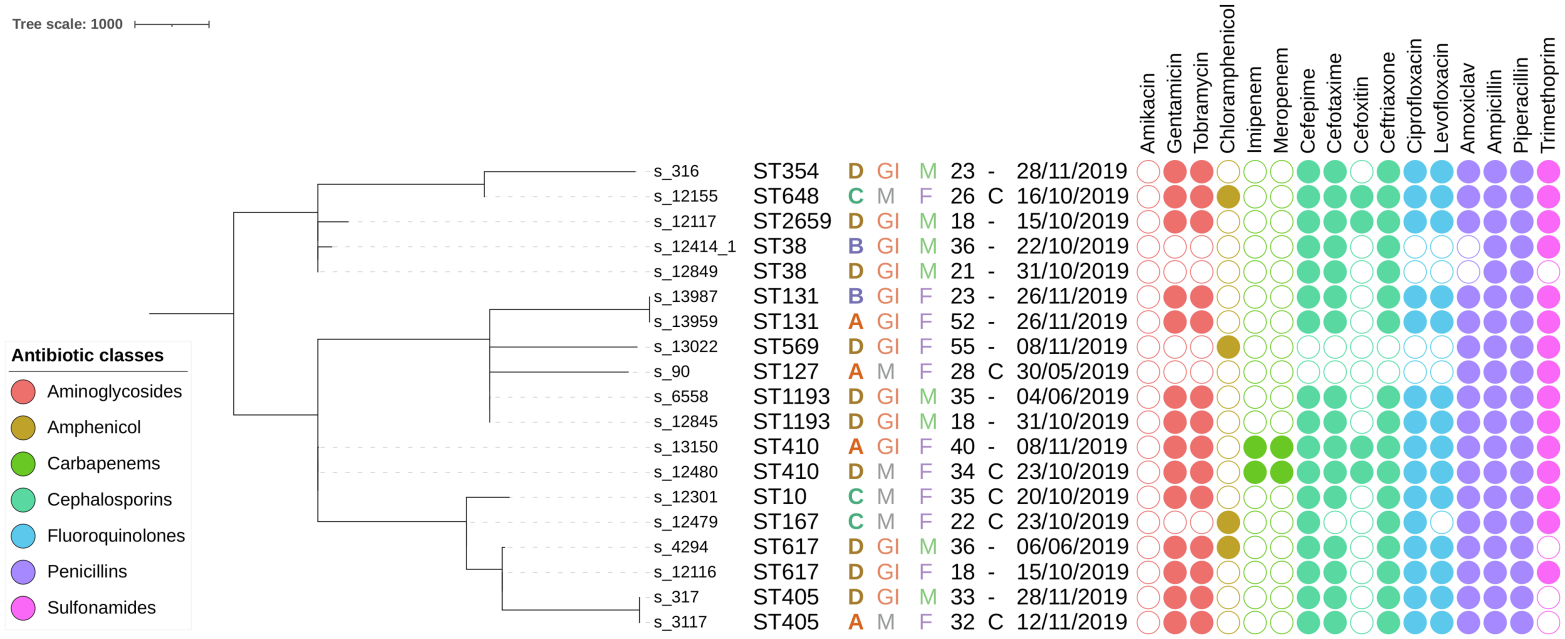
Minimum spanning tree containing all *E. coli* samples sequenced in this study based on cgMLST results. The scale bar expresses the number of allele differences. Annotations are (from left to right) sample name, sequence type (based on classical MLST), hospital letter code, hospital ward, patient gender, patient age, surgical intervention, isolation date, and phenotypically determined AMR susceptibility (a filled circle indicates phenotypic resistance to the corresponding antibiotic). Circles are colored according to the class of the antibiotic as indicated in the legend in the bottom left corner. Abbreviations: maternal (M), gastrointestinal (GI), male (M), female (F), and caesarian (C).

More than 90% of cgMLST loci were detected in all samples from this study. The resulting MST is shown in Figure 2. Generally, considerable phylogenetic differences were observed, with large branch lengths between isolates assigned to different STs. Six groups of two isolates with the same sequence type were found, i.e., ST38, ST131, ST405, ST410, ST617, and ST1193. Samples assigned to ST38 and ST617 differed by 190 and 34 cgMLST alleles, respectively. These relatively large genetic differences indicate that epidemiological links between these cases are unlikely, and the corresponding phylogenies are therefore not discussed in the main manuscript, but a description of the phylogenomic context for these samples can be found in the Supplementary Material. Results for the isolates assigned to the other four STs are described in detail below, in order of decreasing phylogenomic similarity based on cgMLST distance (i.e., cluster with most closely related samples from Benin first). SNP distance matrices for the isolates included in the SNP analysis are provided in Supplementary Tables S9–S12.

### ST405 Cluster

**Figure 3:**
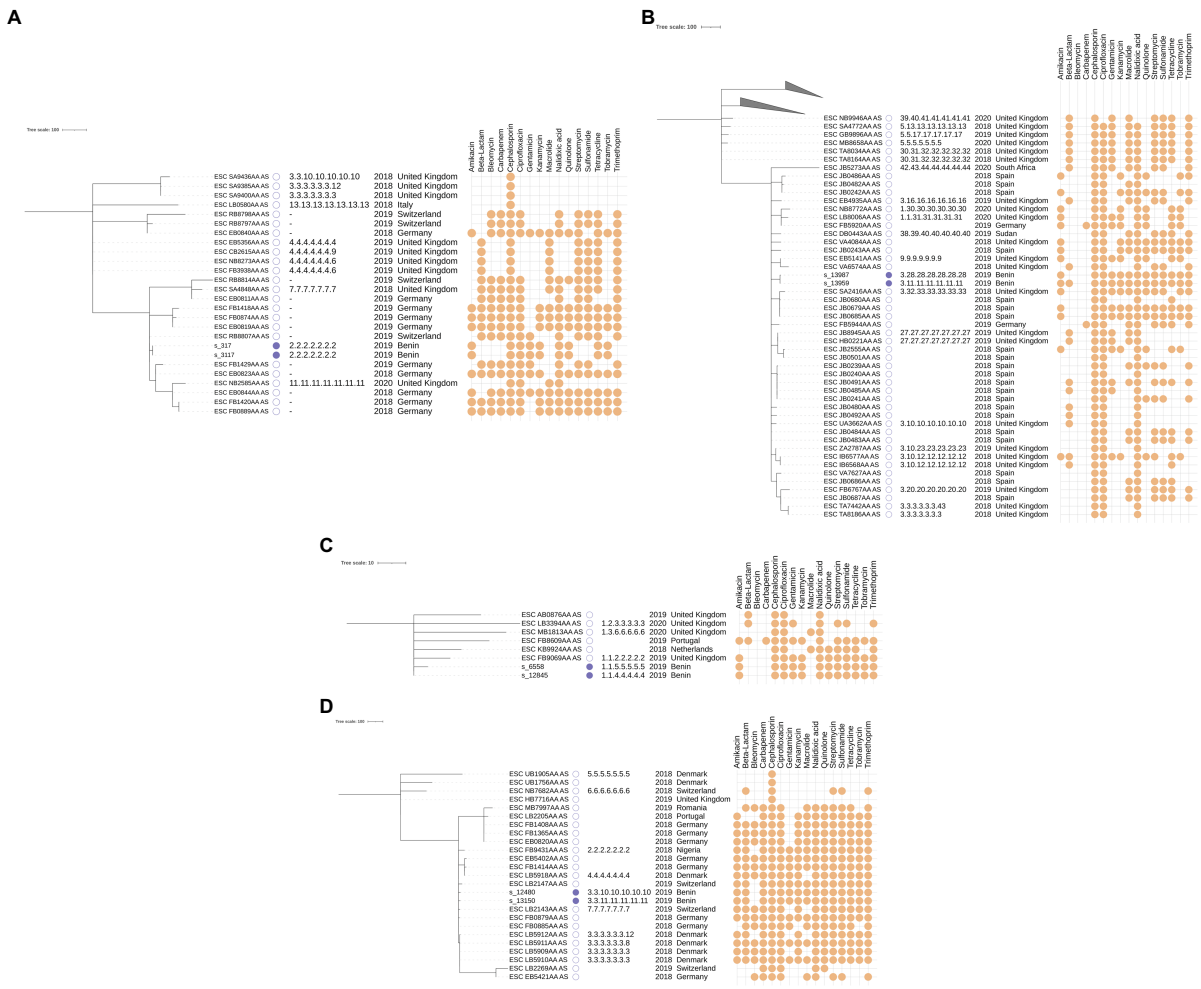
cgMLST-based minimum spanning trees annotated with SNP typing results for samples classified as ST405 **(A)**, ST131 **(B)**, ST1193 **(C)**, and ST410 **(D)**. The scale bar and branch lengths are expressed as the number of allele differences. Annotations are (from left to right) as: sample name, sample origin (this study with a filled blue box or EnteroBase with an empty box), SNP address, isolation year, isolation country, and predicted AMR susceptibility based on genotypic AMR prediction. Samples with missing SNP addresses did not have publicly available Illumina paired-end data or were filtered out because their coverage was too low. Predicted AMR susceptibility is shown for the 16 antibiotics that were most often predicted (full results are provided in Supplementary Table S5). The top two clades in subfigure B were collapsed for visual clarity and contain 18 and 9 isolates, respectively.

The phylogeny for this ST was constructed using 24 additional genomes retrieved from EnteroBase that matched the criteria listed in the Material and Methods. No additional samples from Africa were available. The MST based on cgMLST for this ST is shown in Figure 3A, along with their corresponding SNP addresses. The two samples from this study in this cluster, s_317 and s_3117, were identical based on both cgMLST and SNP typing and exhibited identical phenotypic and genotypic AMR profiles. These results indicate that both isolates are identical, despite being obtained from different patients at different hospitals with 16 days in between. Phenotypic testing of these samples revealed resistance to 8 of the 16 tested antibiotics. The most similar isolate from EnteroBase was ESC_RB8807_AS, collected in Switzerland in 2019, which differed by three cgMLST loci, but WGS read data were not available for this sample rendering it impossible to investigate relatedness based on SNPs.

### ST131 Cluster

The phylogeny for this ST was constructed using an additional 74 genomes retrieved from EnteroBase, which only contained two additional samples from Africa. The MST based on cgMLST and SNP addresses for this ST are shown in Figure 3B. The two samples from this study, s_13987 and s_13959, differed by only three cgMLST loci and had identical phenotypic and similar genotypic AMR profiles (the beta-lactamase gene *bla*_TEM_ was present in only s_13959) but still differed with between 100 and 250 SNPs according to their SNP address. A point source outbreak is therefore unlikely with this amount of observed variation, despite their close time of isolation (both samples were obtained on the same day). Additionally, both samples were obtained in different hospitals. Compared to the samples retrieved from EnteroBase, ESC_JB0483AA_AS isolated in Spain in 2018 was the most closely related isolate but still differed by 47 cgMLST loci. Predicted AMR susceptibility to different antibiotics was widespread in this cluster but did not correlate to the typology of the tree.

### ST1193 Cluster

The phylogeny for this ST was constructed using six additional samples retrieved from EnteroBase. No additional samples from Africa were available. The MST based on cgMLST and SNP addresses for this ST are shown in Figure 3C. The two samples from this study, s_6558 and s_12845, differed by five cgMLST alleles and between 50 and 100 SNPs and exhibited identical phenotypic and genotypic AMR profiles. Both samples were obtained from the same hospital with several months in between (as seen in ST617, described in the Supplementary Results). Similar to the two samples from the ST131 cluster, a point source outbreak appears therefore unlikely.

### ST410 Cluster

The phylogeny for this ST was constructed using 23 additional samples retrieved from EnteroBase. Only one additional sample from Africa was available. The MST based on cgMLST and SNP addresses for this ST are shown in Figure 3D. The two samples from this study, s_12480 and s_13150, differed by 15 cgMLST alleles and between 50 and 100 SNPs. Compared to the samples from this study assigned to other STs, the number of allele differences was quite large compared to the number of SNP differences. Additional screening against inter- and intraspecies contamination did not reveal issues with either sample (results not shown). Both samples exhibited identical phenotypic and genotypic AMR profiles, which both indicated very high rates of resistance. Given the sizable genomic distance between both samples, and the fact they were collected in different hospitals, an epidemiological link between both samples appears unlikely. The only publicly available sample from Africa for this cluster, ESC_FB9431AA_AS, was collected in Nigeria (a neighboring country of Benin) in 2018 but was quite distant from the samples from Benin. The most similar sample from EnteroBase was ESC_FB0885AA_AS, which differed 13 alleles to s_12480 and 12 alleles to s_13150. Since no read data were available for this sample, the SNP address could not be determined.

## Discussion

To the best of our knowledge, this is the first study that employs WGS to investigate AMR profiles and phylogenomic relatedness among clinical *E. coli* isolated in Benin.

All of the 19 selected *E. coli* isolates showed phenotypic resistance to at least four of the 16 phenotypically tested antibiotics, and 14 samples showed resistance to 10 or more. These observations are in line with our previous study conducted in 2019 in the same hospitals, in which we reported 208 MDR-positive samples on a total of 229 aerobic bacteria (90.8%; Yehouenou et al., 2020). High resistance rates were observed in all isolates, especially to aminoglycosides, cephalosporins, penicillins, quinolones, and trimethoprim + sulfamethoxazole. Two isolates were also resistant to carbapenems, which was also observed recently in Nigeria (Aworh et al., 2021), and four were resistant to amphenicol. The high degree of AMR in our study is not surprising, as these antibiotics (ceftriaxone, ciprofloxacin, and beta-lactamases) are easily accessible and commonly used in Benin for therapeutic purposes and very few antimicrobial stewardship programs are in place.

The *bla*_CTX-M-15_ gene is the most frequently reported gene that encodes for CTX-M enzymes in ESBLs (Zhao and Hu, 2013) and was observed in 15 isolates (78.9%) in this study. Four of those isolates also harbored *ampC* genes (*bla*_CMY_) and other beta-lactamase genes (*bla*_TEM-1_). AmpC beta-lactamases are cephalosporinases that are poorly inhibited by clavulanic acid. The co-occurrence of multiple AmpC beta-lactamases was previously reported in *E. coli* in Tunisia (Chérif et al., 2016) and various other studies, especially in isolates collected in African countries (Ribeiro et al., 2016; Sonda et al., 2018; Irenge et al., 2019; Jesumirhewe et al., 2020). These findings suggest that genes from the CTX-M family are currently replacing SHV and TEM in *Enterobacterales*, as reported previously (Camacho et al., 2009).

In our study, 78.9% of ESBL-producing strains (*n* = 15/19) exhibited phenotypic resistance to quinolones, consistent with the previously reported association between ESBL production and quinolone resistance in *Enterobacterales* (Robicsek et al., 2006). This association might be explained by co-selection between ESBL and other AMR genes when exposed to various antimicrobials (Robicsek et al., 2006; Crémet et al., 2011). The *aac*(*6′*)*-Ib-cr* was observed frequently and was present in 14 strains. This gene has spread rapidly among *Enterobacterales*, and although conferring only low-level resistance, it may create an environment that facilitates the selection of highly resistant determinants, especially in organisms harboring other quinolone resistance determinants (Roy et al., 2021).

Out of 62 resistant phenotypes, 60 were correctly genotypically predicted, leading to an accuracy of 82.5%, which is considerably lower than other studies (Kozyreva et al., 2017; Bogaerts et al., 2021). Performance was mainly impacted by lower specificity due to predicted resistances supported only by a single genomic feature, which might not have been sufficient for resistance at the tested dosage. For example, samples s_13022 and s_90 carried the *gyrA* p.S83L mutation, associated with fluoroquinolone resistance, but still were susceptible to the antibiotic. Other samples, such as s_12116 or s_12117, did exhibit phenotypic resistance but also carried three additional mutations associated with resistance to ciprofloxacin. We assume that a similar effect caused the FP predictions for amikacin. This hypothesis is also supported by the fact that our predictions for this antibiotic matched with the results of the NCBI AMRFinderPlus (Feldgarden et al., 2019) and online ResFinder (Zankari et al., 2012) tools (unpublished results). Accuracy increased to 93.6% when the results for amikacin were removed, in line with the performance obtained in the aforementioned studies.

We used MOB-suite to determine the genetic origin of the detected resistance genes as they are known to be frequently or almost exclusively plasmid-encoded (Brolund, 2014), associated with a higher risk of spreading. This analysis confirmed that most of the detected AMR genes were found on contigs predicted to be plasmid-encoded. However, this analysis might have suffered from lower accuracy since only short-read data were available. In future investigations, employing long-read sequencing might therefore be a promising alternative because longs reads can substantially facilitate predicting the genomic origin of detected AMR genes (Lemon et al., 2017; Berbers et al., 2020).

We observed 13 different sequence types with six STs (ST131, ST617, ST38, ST1193, ST410, and ST405) occurring twice, consistent with earlier observations in Tanzania where the same six STs were observed in a collection of 38 *E. coli* samples (Sonda et al., 2018). Resistance to cephalosporins and ciprofloxacin has often been reported in strains classified as ST131 (Cagnacci et al., 2008; Aibinu et al., 2012). The presence of other genetic lineages different from ST131 shows the potential for genetic diversification and emergence of new epidemic strains (Xu et al., 2011). In particular, the association of these clones with quinolone resistance, by carrying the often plasmid-encoded *qnr*, or, *aac*-(*6′*)*-Ib-cr* genes, is very worrisome due to its potential for further dissemination.

Although some STs (e.g., ST410) were found to contain more AMR determinants than other STs (e.g., ST38), AMR profiles were generally not correlated with the phylogeny (see Figure 3), suggesting that horizontal transfer of AMR genes is common, as also shown in numerous other studies on *E. coli* (Rodriguez-Villalobos et al., 2005; Upreti et al., 2018; Nwafia et al., 2019). Nevertheless, some genes have been reported to be associated with particular STs, such as the *bla*_CTX-M-15_ gene, which is often present in the ST131 and ST410 isolates (Tegha et al., 2021), as also observed in this study (Supplementary Table S6).

We observed a generally large diversity among the analyzed *E. coli* strains circulating in the hospitals in Benin. The large phylogenomic differences between the isolates suggest that the infections were caused by multiple generally not very closely related *E. coli* strains, the notable exceptions being samples s_317 and s_3117, which were completely identical. Within individual hospitals, the two most closely related samples differed by at least five cgMLST alleles, i.e., samples s_12845 and s_6558 that were both isolated in hospital D. This diversity was also reflected in the number of observed STs with most samples belonging to different STs and only six STs that contained two isolates. Investigation of the samples assigned to the same STs indicated at least one ST in which both samples were identical (ST405); two STs with samples that were relatively closely related based on cgMLST, with 15 and 5 cgMLST alleles difference, but still differed by between 50 and 100 SNPs (ST410 and ST1193, respectively); and one ST with samples that were closely related based on cgMLST but still differed by between 100 and 250 SNPs (ST131). The sequenced isolates did therefore not indicate a pattern of hospital-acquired and transmitted infections, since they were either identical but isolated in different hospitals (ST405); relatively related but isolated in different hospitals (ST410); or their overall number of SNPs differed by at least 100 (ST38, ST131, ST617, and ST1193). It should be highlighted that these observations do not imply that no hospital-acquired infections and transmissions took place in Benin in 2019, which would be highly unlikely, given that multiple studies have shown that such transmissions constitute a major problem for nosocomial *E. coli* (Peleg and Hooper, 2010; Matta et al., 2018). Most likely, we did not observe such infections and transmissions because not enough samples were isolated and sequenced. More WGS-based screening is required to fully capture the transmission dynamics of the circulating strains. Nevertheless, we did observe a pattern suggestive of sporadic introductions of diverse strains into hospitals from the community, which corroborates results obtained in Tanzania by Sonda et al. in 2018, who observed 21 different sequence types in a collection of 38 clinical *E. coli*, the most common of which were ST131 and ST10 (Sonda et al., 2018). Jesumirhewe et al. observed ST131, ST405, and ST410 in a collection of 17 clinical *E. coli* isolated in Nigeria (Jesumirhewe et al., 2020). ST131 is the dominant international clinical clone, and ST405 was previously associated with the carriage of ESBLs (Aibinu et al., 2012). ST410 has been reported worldwide in extra-intestinal strains associated with resistance to fluoroquinolones, third-generation cephalosporins, and carbapenems (Roer et al., 2018).

Although a few samples in our study were related to isolates sampled in Europe in the background collection retrieved from EnteroBase, only distant phylogenetic relationships could be observed for most of them. This indicates that the background collection is missing isolates to provide the proper phylogenomic context for the samples in our study. This can be explained by the lack of available WGS data from Africa, which accounted for only a very minor fraction of the total dataset (with only two isolates for ST131 and one for ST410 being available). Consequently, the gap in available data and surveillance for Benin and Africa renders it impossible to study the history of the transmission and spread of these *E. coli* isolates within Benin and in a broader African context, and to investigate the relatively close relationship of some samples with European samples. To better understand the spread and dispersion of AMR and pathogenic *E. coli* in Africa, more extensive monitoring in this region, including by WGS, is required.

## Conclusion

We report the first WGS-based analysis of AMR and phylogenomics relatedness of 19 clinical ESBL-positive samples collected from Benin in 2019. Although our study was limited by the relatively low number of sequenced samples, this provides a first genomic resource for Benin. In particular, the observed high levels of *E. coli* diversity regarding their antimicrobial resistance genes and sequence types underline the necessity for concerted efforts to routinely screen more bacterial isolates of clinical importance, even in resource-limited settings, such as Benin. Increased WGS-based surveillance will aid to fill the gaps in observed genotypes and help improve our understanding of the transmission dynamics and the prevalence of AMR genes in *E. coli* within Benin, and by extension, Africa. The information generated in this study not only provides updates on AMR at the hospital level but can also serve as a basis in formulating pragmatic antimicrobial stewardship programs and infection control initiatives, and accentuates the need for the competent authorities to provide more resources to study drug-resistant infections circulating in Benin by means of WGS.

## Data Availability Statement

The datasets supporting the conclusions of this study have been deposited in the NCBI SRA under BioProject PRJNA633966. Metadata and individual accession numbers are available in the Supplementary Material.

## Author Contributions

CY, OD, FD, DA, and AS conceived and designed the study. BB, KV, NR, SK, and KM designed and performed the WGS and bioinformatics analysis. CY, ET, BB, and KV wrote the original draft of the manuscript. All authors contributed to the article and approved the submitted version.

## Funding

This study received financial support from the ARES (Académie de la Recherche pour l’Enseignement Supérieur; Grant number: COOP-CONV-18-108), Belgium.

## Conflict of Interest

The authors declare that the research was conducted in the absence of any commercial or financial relationships that could be construed as a potential conflict of interest.

## Publisher’s Note

All claims expressed in this article are solely those of the authors and do not necessarily represent those of their affiliated organizations, or those of the publisher, the editors and the reviewers. Any product that may be evaluated in this article, or claim that may be made by its manufacturer, is not guaranteed or endorsed by the publisher.

## Footnotes

^1^ https://bitbucket.org/genomicepidemiology/resfinder_db/src/master/antibiotic_classes.txt

^2^ https://github.com/phe-bioinformatics/PHEnix
