# Supplementary material for: Whole-Genome Sequencing-Based Antimicrobial Resistance Characterization and Phylogenomic Investigation of 19 Multidrug-Resistant and Extended-Spectrum Beta-Lactamase-Positive Escherichia coli Strains Collected From Hospital Patients in Benin in 2019
Source: Front Microbiol. 2021 Dec 9;12:752883. doi: 10.3389/fmicb.2021.752883 (PMC8695880; doi:10.3389/fmicb.2021.752883)
Supplement: Supplementary file 1 [file Data_Sheet_1.docx]

# **Supplementary Material**

## **Supplementary Results**

### **ST38 cluster**

The phylogeny for this ST was constructed using 139 samples retrieved from EnteroBase that matched the criteria listed in the Material and Methods. The MST based on cgMLST for this ST is shown in Figure S1a, along with their corresponding SNP addresses. Compared to the other STs discussed below, the variation between samples from this ST was the largest. Predicted resistance for samples in this ST was generally lower than for other STs. Both in-house samples were not closely related and differed by 190 cgMLST loci and 250 SNPs of each other (Figure 2a). Given the large genomic distance between both samples, and the fact that they were collected in different hospitals, a link between both samples is unlikely. The most closely related sample from EnteroBase for both samples was ESC_UB3248AA_AS, collected in Spain in 2020. This sample differed nine alleles with sample s_12849 and 207 alleles with sample 12414_1.

### **ST617 cluster**

The phylogeny for this ST was constructed using six samples retrieved from EnteroBase that matched the criteria listed in the Material and Methods. The MST based on cgMLST for this ST is shown in Figure S1b, along with their corresponding SNP addresses. No other samples with this ST originating from Africa were available in EnteroBase. The MST based on cgMLST for this ST is shown in Figure S1b, along with SNP addresses that were added as annotations. Relatively large differences were observed between both samples from this study, s_4294 and s_12116, which differed by 35 cgMLST loci and over 250 SNPs. Both samples were collected at the same hospital with approximately four months in between, but the large overall distance suggests that both samples are unrelated to each other. The most closely related sample retrieved from NCBI was ESC_TA8108AA_AS, isolated in the United Kingdom in 2018, but this sample still differed by 62 alleles and over 250 SNPs.

### **Analysis of the origin of the reads classified as *Acinetobacter* by Kraken 2**

We extracted and *de novo* assembled all reads assigned to *Acinetobacter* at the genus level, resulting in assemblies with N50 of 27,382 and 47,939 for samples s_316 and s_4294, respectively. Sequence typing was performed using the ribosomal MLST scheme, using the same approach as the cgMLST analysis described in the main manuscript. This analysis revealed the presence of 52 (98.11%) in sample s_316 and 53 (100%) in sample s_4294 of the rMLST loci in the assembled contigs, matching with ribosomal sequence type 8482, annotated as *Acinetobacter buamannii*. Since all of these loci are typically located on the chromosome, this strongly suggests the presence of an *Acinetobacter* strain, instead of solely a plasmid. Additionally, we mapped the reads classified as *Acinetobacter* to the *A. baumannii* reference genome ([NZ_CP043953.1](https://www.ncbi.nlm.nih.gov/nuccore/NZ_CP043953.1)) using Bowtie 2 (version 2.4.1) with default settings, resulting in 84.63% and 84.81% of positions in the reference genome that were covered by at least one read for the filtered reads from samples s_316 and s_4294, respectively. These findings suggest that the contamination indicated by the Kraken 2 analysis was most likely caused by the presence of a strain and not only a plasmid.

## **Supplementary Tables**

### **Table S1: Accession numbers**

| **sample** | **SRA accession** |
| --- | --- |
| s_12116 | SRR13687105 |
| s_12117 | SRR13687097 |
| s_12155 | SRR13687103 |
| s_12301 | SRR13687113 |
| s_12414 | SRR13687095 |
| s_12479 | SRR13687098 |
| s_12480 | SRR13687108 |
| s_12845 | SRR13687112 |
| s_12849 | SRR13687111 |
| s_13022 | SRR13687106 |
| s_13150 | SRR13687107 |
| s_13959 | SRR13687100 |
| s_13987 | SRR13687099 |
| s_3117 | SRR13687110 |
| s_316 | SRR13687096 |
| s_317 | SRR13687109 |
| s_4294 | SRR13687104 |
| s_6558 | SRR13687102 |
| s_90 | SRR13687101 |

The first column lists the sample name, the second column the SRA accession number.

### **Table S2: Reference genomes used for SNP typing for the different STs.**

| **ST** | **Reference genome** |
| --- | --- |
| ST38 | ESC_LB2393AA_AS |
| ST131 | ESC_LB2114AA_AS |
| ST405 | ESC_LB2126AA_AS |
| ST410 | ESC_GA4687AA_AS |
| ST617 | ESC_VA2053AA_AS |
| ST1193 | ESC_WA1759AA_AS |

The accession for the reference genome refers to the EnteroBase nomenclature. Abbreviations: ST (Sequence Type).

### **Table S3: Read-trimming statistics.**

| **Sample** | **Read pairs** | **Both surviving** | **Forward only surviving** | **Reverse only surviving** | **Both dropped** |
| --- | --- | --- | --- | --- | --- |
| s_12116 | 1,017,876 | 940,995 | 62,206 | 5,852 | 8,823 |
| s_12117 | 719,360 | 655,114 | 55,739 | 2,741 | 5,766 |
| s_12155 | 587,329 | 538,578 | 41,951 | 2,055 | 4,745 |
| s_12301 | 481,603 | 403,464 | 69,747 | 1,569 | 6,823 |
| s_12414_1 | 958,454 | 870,512 | 75,245 | 4,364 | 8,333 |
| s_12479 | 467,136 | 415,172 | 45,659 | 1,528 | 4,777 |
| s_12480 | 989,171 | 921,512 | 54,801 | 4,961 | 7,897 |
| s_12845 | 801,385 | 751,532 | 38,919 | 5,024 | 5,910 |
| s_12849 | 681,780 | 608,004 | 65,076 | 2,311 | 6,389 |
| s_13022 | 506,466 | 454,185 | 46,217 | 1,847 | 4,217 |
| s_13150 | 1,108,148 | 1,039,625 | 52,800 | 7,519 | 8,204 |
| s_13959 | 333,833 | 289,062 | 40,532 | 828 | 3,411 |
| s_13987 | 854,096 | 790,656 | 51,765 | 4,889 | 6,786 |
| s_3117 | 920,439 | 839,346 | 69,738 | 3,853 | 7,502 |
| s_316* | 858,923 | 788,587 | 59,465 | 4,023 | 6,848 |
| s_317 | 791,104 | 721,629 | 59,245 | 3,700 | 6,530 |
| s_4294* | 897,220 | 821,420 | 64,572 | 4,102 | 7,126 |
| s_6558 | 786,953 | 723,038 | 51,580 | 5,628 | 6,707 |
| s_90 | 734,084 | 666,102 | 58,438 | 3,171 | 6,373 |

The first and second columns list the sample name and the total number of read pairs, respectively. The third, fourth, fifth and sixth columns list the number of read pairs where both members of the read pair passed trimming, the number of read pairs where only the forward read passed read trimming, the number of read pairs where only the reverse read passed read trimming, and the number of read pairs where both read pair members were dropped in the trimming step. (*) A fraction of reads for samples s_316 and s_4294 were assigned to the *Acinetobacter* genus and were discarded afterwards.

### **Table S4: Assembly statistics.**

| **Sample** | **Nb. of contigs** | **N50** | **Total genome assembly length** |
| --- | --- | --- | --- |
| s_12116 | 125 | 126,606 | 5,045,411 |
| s_12117 | 93 | 128,454 | 5,151,059 |
| s_12155 | 95 | 153,279 | 5,064,374 |
| s_12301 | 73 | 168,657 | 4,938,064 |
| s_12414_1 | 68 | 206,691 | 5,054,038 |
| s_12479 | 95 | 139,671 | 4,905,402 |
| s_12480 | 72 | 174,413 | 4,930,133 |
| s_12845 | 61 | 237,339 | 5,007,359 |
| s_12849 | 43 | 259,445 | 4,882,819 |
| s_13022 | 58 | 314,276 | 4,950,715 |
| s_13150 | 349 | 137,393 | 5,412,554 |
| s_13959 | 76 | 203,646 | 5,290,864 |
| s_13987 | 68 | 206,216 | 5,318,453 |
| s_3117 | 166 | 100,882 | 5,513,901 |
| s_316* | 665 | 28,361 | 6,385,055 |
| s_317 | 117 | 108,482 | 5,412,664 |
| s_4294* | 289 | 113,855 | 4,836,832 |
| s_6558 | 91 | 375,440 | 5,132,256 |
| s_90 | 50 | 288,389 | 5,198,767 |

The first column lists the sample name. The second, third, and fourth columns list the number of contigs, N50, and total assembly length, respectively. * Sample s_316 and s_4294 were assembled after removing reads assigned to the *Acinetobacter* genus.

### **Table S5: Detected sequence types.**

| **Sample** | **Sequence type** |
| --- | --- |
| s_12301 | ST10 |
| s_12845 | ST1193 |
| s_6558 | ST1193 |
| s_90 | ST127 |
| s_13959 | ST131 |
| s_13987 | ST131 |
| s_12479 | ST167 |
| s_12117 | ST2659 |
| s_316 | ST354 |
| s_12414 | ST38 |
| s_12849 | ST38 |
| s_3117 | ST405 |
| s_317 | ST405 |
| s_12480 | ST410 |
| s_13150 | ST410 |
| s_13022 | ST569 |
| s_12116 | ST617 |
| s_4294 | ST617 |
| s_12155 | ST648 |

The first column lists the sample name, the second column lists the detected sequence type.

### **Table S6: Detected AMR genes and mutations.**

| **Sample** | **Antibiotic(s)** | **AMR genes** | **Mutations** |
| --- | --- | --- | --- |
| s_12116 | Amikacin | aac(6')-Ib-D181Y_aac(6')-Ib-W104R_aac(6')-Ib-cr_aac(6')-Ib-cr10_aac(6')-Ib-cr11_aac(6')-Ib-cr4_aac(6')-Ib-cr5 | - |
| s_12117 | Amikacin | aac(6')-Ib-D181Y_aac(6')-Ib-W104R_aac(6')-Ib-cr_aac(6')-Ib-cr10_aac(6')-Ib-cr11_aac(6')-Ib-cr4_aac(6')-Ib-cr5 | - |
| s_12155 | Amikacin | aac(6')-Ib-D181Y_aac(6')-Ib-W104R_aac(6')-Ib-cr_aac(6')-Ib-cr10_aac(6')-Ib-cr11_aac(6')-Ib-cr4_aac(6')-Ib-cr5 | - |
| s_12301 | Amikacin | aac(6')-Ib-D181Y_aac(6')-Ib-W104R_aac(6')-Ib-cr_aac(6')-Ib-cr10_aac(6')-Ib-cr11_aac(6')-Ib-cr4_aac(6')-Ib-cr5 | - |
| s_12414 | Amikacin | - | - |
| s_12479 | Amikacin | aac(6')-Ib-D181Y_aac(6')-Ib-W104R_aac(6')-Ib-cr_aac(6')-Ib-cr10_aac(6')-Ib-cr11_aac(6')-Ib-cr4_aac(6')-Ib-cr5 | - |
| s_12480 | Amikacin | aac(6')-Ib-D181Y_aac(6')-Ib-W104R_aac(6')-Ib-cr_aac(6')-Ib-cr10_aac(6')-Ib-cr11_aac(6')-Ib-cr4_aac(6')-Ib-cr5 | - |
| s_12845 | Amikacin | aac(6')-Ib-D181Y_aac(6')-Ib-W104R_aac(6')-Ib-cr_aac(6')-Ib-cr10_aac(6')-Ib-cr11_aac(6')-Ib-cr4_aac(6')-Ib-cr5 | - |
| s_12849 | Amikacin | - | - |
| s_13022 | Amikacin | - | - |
| s_13150 | Amikacin | aac(6')-Ib-D181Y_aac(6')-Ib-W104R_aac(6')-Ib-cr_aac(6')-Ib-cr10_aac(6')-Ib-cr11_aac(6')-Ib-cr4_aac(6')-Ib-cr5 | - |
| s_13959 | Amikacin | aac(6')-Ib-D181Y_aac(6')-Ib-W104R_aac(6')-Ib-cr_aac(6')-Ib-cr10_aac(6')-Ib-cr11_aac(6')-Ib-cr4_aac(6')-Ib-cr5 | - |
| s_13987 | Amikacin | aac(6')-Ib-D181Y_aac(6')-Ib-W104R_aac(6')-Ib-cr_aac(6')-Ib-cr10_aac(6')-Ib-cr11_aac(6')-Ib-cr4_aac(6')-Ib-cr5 | - |
| s_3117 | Amikacin | aac(6')-Ib-D181Y_aac(6')-Ib-W104R_aac(6')-Ib-cr_aac(6')-Ib-cr10_aac(6')-Ib-cr11_aac(6')-Ib-cr4_aac(6')-Ib-cr5 | - |
| s_316 | Amikacin | aac(6')-Ib-D181Y_aac(6')-Ib-W104R_aac(6')-Ib-cr_aac(6')-Ib-cr10_aac(6')-Ib-cr11_aac(6')-Ib-cr4_aac(6')-Ib-cr5 | - |
| s_317 | Amikacin | aac(6')-Ib-D181Y_aac(6')-Ib-W104R_aac(6')-Ib-cr_aac(6')-Ib-cr10_aac(6')-Ib-cr11_aac(6')-Ib-cr4_aac(6')-Ib-cr5 | - |
| s_4294 | Amikacin | - | - |
| s_6558 | Amikacin | aac(6')-Ib-D181Y_aac(6')-Ib-W104R_aac(6')-Ib-cr_aac(6')-Ib-cr10_aac(6')-Ib-cr11_aac(6')-Ib-cr4_aac(6')-Ib-cr5 | - |
| s_90 | Amikacin | - | - |
| s_12116 | Beta-Lactam | - | - |
| s_12117 | Beta-Lactam | - | - |
| s_12155 | Beta-Lactam | blaTEM-1_blaTEM-235_blaTEM-40 | - |
| s_12301 | Beta-Lactam | blaEC-15_blaEC-8_blaEC-19_blaEC_blaEC-5 | - |
| s_12411 | Beta-Lactam | blaTEM-1_blaTEM-235_blaTEM-40 | - |
| s_12479 | Beta-Lactam | - | - |
| s_12480 | Beta-Lactam | blaTEM-1_blaTEM-235_blaTEM-40 | - |
| s_12845 | Beta-Lactam | - | - |
| s_12849 | Beta-Lactam | - | - |
| s_13022 | Beta-Lactam | blaTEM-1_blaTEM-235_blaTEM-40 | - |
| s_13150 | Beta-Lactam | blaTEM-1_blaTEM-235_blaTEM-40 | - |
| s_13959 | Beta-Lactam | blaTEM-1_blaTEM-235_blaTEM-40 | - |
| s_13987 | Beta-Lactam | - | - |
| s_3117 | Beta-Lactam | - | - |
| s_316 | Beta-Lactam | blaTEM-1_blaTEM-235_blaTEM-40 | - |
| s_317 | Beta-Lactam | - | - |
| s_4294 | Beta-Lactam | blaTEM-1_blaTEM-235_blaTEM-40 | - |
| s_6558 | Beta-Lactam | - | - |
| s_90 | Beta-Lactam | blaTEM-1_blaTEM-235_blaTEM-40 | - |
| s_12116 | Carbapenem | - | - |
| s_12117 | Carbapenem | - | - |
| s_12155 | Carbapenem | - | - |
| s_12301 | Carbapenem | - | - |
| s_12414 | Carbapenem | - | - |
| s_12479 | Carbapenem | - | - |
| s_12480 | Carbapenem | blaOXA-181 | - |
| s_12845 | Carbapenem | - | - |
| s_12849 | Carbapenem | - | - |
| s_13022 | Carbapenem | - | - |
| s_13150 | Carbapenem | blaOXA-181 | - |
| s_13959 | Carbapenem | - | - |
| s_13987 | Carbapenem | - | - |
| s_3117 | Carbapenem | - | - |
| s_316 | Carbapenem | - | - |
| s_317 | Carbapenem | - | - |
| s_4294 | Carbapenem | - | - |
| s_6558 | Carbapenem | - | - |
| s_90 | Carbapenem | - | - |
| s_12116 | Cephalosporin | blaOXA-1, blaCTX-M-15, blaEC-15_blaEC-8_blaEC-19_blaEC_blaEC-5 | - |
| s_12117 | Cephalosporin | blaCMY-42_blaCMY-2, blaOXA-1, blaCTX-M-15, blaEC-15_blaEC-8_blaEC-19_blaEC_blaEC-5 | - |
| s_12155 | Cephalosporin | blaOXA-1, blaCTX-M-15, blaEC-15_blaEC-8_blaEC-19_blaEC_blaEC-5 | - |
| s_12301 | Cephalosporin | blaOXA-1, blaCTX-M-15 | - |
| s_12414 | Cephalosporin | blaCTX-M-15, blaEC-15_blaEC-8_blaEC-19_blaEC_blaEC-5 | - |
| s_12479 | Cephalosporin | blaOXA-1, blaCTX-M-15, blaEC-15_blaEC-8_blaEC-19_blaEC_blaEC-5 | - |
| s_12480 | Cephalosporin | blaCMY-42_blaCMY-2, blaOXA-1, blaCTX-M-15, blaEC-15_blaEC-8_blaEC-19_blaEC_blaEC-5 | - |
| s_12845 | Cephalosporin | blaOXA-1, blaCTX-M-15, blaEC-15_blaEC-8_blaEC-19_blaEC_blaEC-5 | - |
| s_12849 | Cephalosporin | blaCTX-M-15, blaEC-15_blaEC-8_blaEC-19_blaEC_blaEC-5 | - |
| s_13022 | Cephalosporin | blaEC-15_blaEC-8_blaEC-19_blaEC_blaEC-5 | - |
| s_13150 | Cephalosporin | blaCMY-42_blaCMY-2, blaOXA-1, blaCTX-M-15, blaEC-15_blaEC-8_blaEC-19_blaEC_blaEC-5 | - |
| s_13959 | Cephalosporin | blaOXA-1, blaCTX-M-15, blaEC-15_blaEC-8_blaEC-19_blaEC_blaEC-5 | - |
| s_13987 | Cephalosporin | blaOXA-1, blaCTX-M-15, blaEC-15_blaEC-8_blaEC-19_blaEC_blaEC-5 | - |
| s_3117 | Cephalosporin | blaCMY-42_blaCMY-2, blaOXA-1, blaEC-15_blaEC-8_blaEC-19_blaEC_blaEC-5 | - |
| s_316 | Cephalosporin | blaOXA-1, blaCTX-M-15, blaEC-15_blaEC-8_blaEC-19_blaEC_blaEC-5 | - |
| s_317 | Cephalosporin | blaCMY-42_blaCMY-2, blaOXA-1, blaEC-15_blaEC-8_blaEC-19_blaEC_blaEC-5 | - |
| s_4294 | Cephalosporin | blaCTX-M-15, blaEC-15_blaEC-8_blaEC-19_blaEC_blaEC-5 | - |
| s_6558 | Cephalosporin | blaOXA-1, blaCTX-M-15, blaEC-15_blaEC-8_blaEC-19_blaEC_blaEC-5 | - |
| s_90 | Cephalosporin | blaEC-15_blaEC-8_blaEC-19_blaEC_blaEC-5 | - |
| s_12116 | Chloramphenicol | - | - |
| s_12117 | Chloramphenicol | - | - |
| s_12155 | Chloramphenicol | catA1 | - |
| s_12301 | Chloramphenicol | - | - |
| s_12414 | Chloramphenicol | - | - |
| s_12479 | Chloramphenicol | catA1 | - |
| s_12480 | Chloramphenicol | - | - |
| s_12845 | Chloramphenicol | - | - |
| s_12849 | Chloramphenicol | - | - |
| s_13022 | Chloramphenicol | catA1 | - |
| s_13150 | Chloramphenicol | - | - |
| s_13959 | Chloramphenicol | - | - |
| s_13987 | Chloramphenicol | - | - |
| s_3117 | Chloramphenicol | - | - |
| s_316 | Chloramphenicol | - | - |
| s_317 | Chloramphenicol | - | - |
| s_4294 | Chloramphenicol | catA1 | - |
| s_6558 | Chloramphenicol | - | - |
| s_90 | Chloramphenicol | - | - |
| s_12116 | Ciprofloxacin | - | gyrA p.S83L, gyrA p.D87N, parC p.S80I, parE p.S458A |
| s_12117 | Ciprofloxacin | - | gyrA p.S83L, gyrA p.D87N, parC p.S80I, parE p.S458A |
| s_12155 | Ciprofloxacin | - | gyrA p.S83L, gyrA p.D87N, parC p.S80I, parE p.S458A |
| s_12301 | Ciprofloxacin | - | gyrA p.S83L, gyrA p.D87N, parC p.S80I, parE p.L416F |
| s_12414 | Ciprofloxacin | - | - |
| s_12479 | Ciprofloxacin | - | gyrA p.S83L, gyrA p.D87N, parC p.S80I, parE p.S458A |
| s_12480 | Ciprofloxacin | - | gyrA p.S83L, gyrA p.D87N, parC p.S80I, parE p.S458A |
| s_12845 | Ciprofloxacin | - | gyrA p.S83L, gyrA p.D87N, parC p.S80I, parE p.L416F |
| s_12849 | Ciprofloxacin | - | - |
| s_13022 | Ciprofloxacin | - | gyrA p.S83L |
| s_13150 | Ciprofloxacin | - | gyrA p.S83L, gyrA p.D87N, parC p.S80I, parE p.S458A |
| s_13959 | Ciprofloxacin | - | gyrA p.S83L, gyrA p.D87N, parC p.S80I, parC p.E84V, parE p.I529L |
| s_13987 | Ciprofloxacin | - | gyrA p.S83L, gyrA p.D87N, parC p.S80I, parC p.E84V, parE p.I529L |
| s_3117 | Ciprofloxacin | - | gyrA p.S83L, gyrA p.D87N, parC p.S80I, parE p.S458A |
| s_316 | Ciprofloxacin | - | gyrA p.S83L, gyrA p.D87N, parC p.S80I, parC p.E84G, parE p.I355T |
| s_317 | Ciprofloxacin | - | gyrA p.S83L, gyrA p.D87N, parC p.S80I, parE p.S458A |
| s_4294 | Ciprofloxacin | - | gyrA p.S83L, gyrA p.D87N, parC p.S80I, parE p.S458A |
| s_6558 | Ciprofloxacin | - | gyrA p.S83L, gyrA p.D87N, parC p.S80I, parE p.L416F |
| s_90 | Ciprofloxacin | - | gyrA p.S83L |
| s_12116 | Gentamicin | aac(3)-IIe_aac(3)-IId | - |
| s_12117 | Gentamicin | - | - |
| s_12155 | Gentamicin | aac(3)-IIe_aac(3)-IId | - |
| s_12301 | Gentamicin | aac(3)-IIe_aac(3)-IId | - |
| s_12414 | Gentamicin | - | - |
| s_12479 | Gentamicin | - | - |
| s_12480 | Gentamicin | aac(3)-IIe_aac(3)-IId | - |
| s_12845 | Gentamicin | aac(3)-IIe_aac(3)-IId | - |
| s_12849 | Gentamicin | - | - |
| s_13022 | Gentamicin | - | - |
| s_13150 | Gentamicin | aac(3)-IIe_aac(3)-IId | - |
| s_13959 | Gentamicin | aac(3)-IIe_aac(3)-IId | - |
| s_13987 | Gentamicin | aac(3)-IIe_aac(3)-IId | - |
| s_3117 | Gentamicin | aac(3)-IIe_aac(3)-IId | - |
| s_316 | Gentamicin | aac(3)-IIe_aac(3)-IId, aac(3)-Ia | - |
| s_317 | Gentamicin | aac(3)-IIe_aac(3)-IId | - |
| s_4294 | Gentamicin | aac(3)-IIe_aac(3)-IId, aac(3)-Ia | - |
| s_6558 | Gentamicin | aac(3)-IIe_aac(3)-IId | - |
| s_90 | Gentamicin | - | - |
| s_12116 | Kanamycin | aac(6')-Ib-D181Y_aac(6')-Ib-W104R_aac(6')-Ib-cr_aac(6')-Ib-cr10_aac(6')-Ib-cr11_aac(6')-Ib-cr4_aac(6')-Ib-cr5 | - |
| s_12117 | Kanamycin | aac(6')-Ib-D181Y_aac(6')-Ib-W104R_aac(6')-Ib-cr_aac(6')-Ib-cr10_aac(6')-Ib-cr11_aac(6')-Ib-cr4_aac(6')-Ib-cr5 | - |
| s_12155 | Kanamycin | aac(6')-Ib-D181Y_aac(6')-Ib-W104R_aac(6')-Ib-cr_aac(6')-Ib-cr10_aac(6')-Ib-cr11_aac(6')-Ib-cr4_aac(6')-Ib-cr5 | - |
| s_12301 | Kanamycin | aac(6')-Ib-D181Y_aac(6')-Ib-W104R_aac(6')-Ib-cr_aac(6')-Ib-cr10_aac(6')-Ib-cr11_aac(6')-Ib-cr4_aac(6')-Ib-cr5 | - |
| s_12414 | Kanamycin | - | - |
| s_12479 | Kanamycin | aac(6')-Ib-D181Y_aac(6')-Ib-W104R_aac(6')-Ib-cr_aac(6')-Ib-cr10_aac(6')-Ib-cr11_aac(6')-Ib-cr4_aac(6')-Ib-cr5 | - |
| s_12480 | Kanamycin | aac(6')-Ib-D181Y_aac(6')-Ib-W104R_aac(6')-Ib-cr_aac(6')-Ib-cr10_aac(6')-Ib-cr11_aac(6')-Ib-cr4_aac(6')-Ib-cr5 | - |
| s_12845 | Kanamycin | aac(6')-Ib-D181Y_aac(6')-Ib-W104R_aac(6')-Ib-cr_aac(6')-Ib-cr10_aac(6')-Ib-cr11_aac(6')-Ib-cr4_aac(6')-Ib-cr5 | - |
| s_12849 | Kanamycin | - | - |
| s_13022 | Kanamycin | - | - |
| s_13150 | Kanamycin | aac(6')-Ib-D181Y_aac(6')-Ib-W104R_aac(6')-Ib-cr_aac(6')-Ib-cr10_aac(6')-Ib-cr11_aac(6')-Ib-cr4_aac(6')-Ib-cr5 | - |
| s_13959 | Kanamycin | aac(6')-Ib-D181Y_aac(6')-Ib-W104R_aac(6')-Ib-cr_aac(6')-Ib-cr10_aac(6')-Ib-cr11_aac(6')-Ib-cr4_aac(6')-Ib-cr5 | - |
| s_13987 | Kanamycin | aac(6')-Ib-D181Y_aac(6')-Ib-W104R_aac(6')-Ib-cr_aac(6')-Ib-cr10_aac(6')-Ib-cr11_aac(6')-Ib-cr4_aac(6')-Ib-cr5 | - |
| s_3117 | Kanamycin | aac(6')-Ib-D181Y_aac(6')-Ib-W104R_aac(6')-Ib-cr_aac(6')-Ib-cr10_aac(6')-Ib-cr11_aac(6')-Ib-cr4_aac(6')-Ib-cr5 | - |
| s_316 | Kanamycin | aac(6')-Ib-D181Y_aac(6')-Ib-W104R_aac(6')-Ib-cr_aac(6')-Ib-cr10_aac(6')-Ib-cr11_aac(6')-Ib-cr4_aac(6')-Ib-cr5 | - |
| s_317 | Kanamycin | aac(6')-Ib-D181Y_aac(6')-Ib-W104R_aac(6')-Ib-cr_aac(6')-Ib-cr10_aac(6')-Ib-cr11_aac(6')-Ib-cr4_aac(6')-Ib-cr5 | - |
| s_4294 | Kanamycin | - | - |
| s_6558 | Kanamycin | aac(6')-Ib-D181Y_aac(6')-Ib-W104R_aac(6')-Ib-cr_aac(6')-Ib-cr10_aac(6')-Ib-cr11_aac(6')-Ib-cr4_aac(6')-Ib-cr5 | - |
| s_90 | Kanamycin | - | - |
| s_12116 | Macrolide | mph(A) | - |
| s_12117 | Macrolide | mph(A), erm(B) | - |
| s_12155 | Macrolide | mph(A) | - |
| s_12301 | Macrolide | mph(A) | - |
| s_12414 | Macrolide | - | - |
| s_12479 | Macrolide | mph(A) | - |
| s_12480 | Macrolide | mph(A) | - |
| s_12845 | Macrolide | - | - |
| s_12849 | Macrolide | - | - |
| s_13022 | Macrolide | mph(A) | - |
| s_13150 | Macrolide | mph(A) | - |
| s_13959 | Macrolide | mph(A) | - |
| s_13987 | Macrolide | mph(A) | - |
| s_3117 | Macrolide | - | - |
| s_316 | Macrolide | - | - |
| s_317 | Macrolide | - | - |
| s_4294 | Macrolide | mph(A) | - |
| s_6558 | Macrolide | - | - |
| s_90 | Macrolide | - | - |
| s_12116 | Nalidixic acid | - | gyrA p.S83L, gyrA p.D87N, parC p.S80I, parE p.S458A |
| s_12117 | Nalidixic acid | - | gyrA p.S83L, gyrA p.D87N, parC p.S80I, parE p.S458A |
| s_12155 | Nalidixic acid | - | gyrA p.S83L, gyrA p.D87N, parC p.S80I, parE p.S458A |
| s_12301 | Nalidixic acid | - | gyrA p.S83L, gyrA p.D87N, parC p.S80I, parE p.L416F |
| s_12414 | Nalidixic acid | - | - |
| s_12479 | Nalidixic acid | - | gyrA p.S83L, gyrA p.D87N, parC p.S80I, parE p.S458A |
| s_12480 | Nalidixic acid | - | gyrA p.S83L, gyrA p.D87N, parC p.S80I, parE p.S458A |
| s_12845 | Nalidixic acid | - | gyrA p.S83L, gyrA p.D87N, parC p.S80I, parE p.L416F |
| s_12849 | Nalidixic acid | - | - |
| s_13022 | Nalidixic acid | - | gyrA p.S83L |
| s_13150 | Nalidixic acid | - | gyrA p.S83L, gyrA p.D87N, parC p.S80I, parE p.S458A |
| s_13959 | Nalidixic acid | - | gyrA p.S83L, gyrA p.D87N, parC p.S80I, parC p.E84V, parE p.I529L |
| s_13987 | Nalidixic acid | - | gyrA p.S83L, gyrA p.D87N, parC p.S80I, parC p.E84V, parE p.I529L |
| s_3117 | Nalidixic acid | - | gyrA p.S83L, gyrA p.D87N, parC p.S80I, parE p.S458A |
| s_316 | Nalidixic acid | - | gyrA p.S83L, gyrA p.D87N, parC p.S80I, parC p.E84G, parE p.I355T |
| s_317 | Nalidixic acid | - | gyrA p.S83L, gyrA p.D87N, parC p.S80I, parE p.S458A |
| s_4294 | Nalidixic acid | - | gyrA p.S83L, gyrA p.D87N, parC p.S80I, parE p.S458A |
| s_6558 | Nalidixic acid | - | gyrA p.S83L, gyrA p.D87N, parC p.S80I, parE p.L416F |
| s_90 | Nalidixic acid | - | gyrA p.S83L |
| s_12116 | Quinolone | aac(6')-Ib-D181Y_aac(6')-Ib-W104R_aac(6')-Ib-cr_aac(6')-Ib-cr10_aac(6')-Ib-cr11_aac(6')-Ib-cr4_aac(6')-Ib-cr5 | - |
| s_12117 | Quinolone | aac(6')-Ib-D181Y_aac(6')-Ib-W104R_aac(6')-Ib-cr_aac(6')-Ib-cr10_aac(6')-Ib-cr11_aac(6')-Ib-cr4_aac(6')-Ib-cr5 | - |
| s_12155 | Quinolone | aac(6')-Ib-D181Y_aac(6')-Ib-W104R_aac(6')-Ib-cr_aac(6')-Ib-cr10_aac(6')-Ib-cr11_aac(6')-Ib-cr4_aac(6')-Ib-cr5 | - |
| s_12301 | Quinolone | aac(6')-Ib-D181Y_aac(6')-Ib-W104R_aac(6')-Ib-cr_aac(6')-Ib-cr10_aac(6')-Ib-cr11_aac(6')-Ib-cr4_aac(6')-Ib-cr5 | - |
| s_12414 | Quinolone | qnrS1 | - |
| s_12479 | Quinolone | aac(6')-Ib-D181Y_aac(6')-Ib-W104R_aac(6')-Ib-cr_aac(6')-Ib-cr10_aac(6')-Ib-cr11_aac(6')-Ib-cr4_aac(6')-Ib-cr5 | - |
| s_12480 | Quinolone | qnrS1, aac(6')-Ib-D181Y_aac(6')-Ib-W104R_aac(6')-Ib-cr_aac(6')-Ib-cr10_aac(6')-Ib-cr11_aac(6')-Ib-cr4_aac(6')-Ib-cr5 | - |
| s_12845 | Quinolone | aac(6')-Ib-D181Y_aac(6')-Ib-W104R_aac(6')-Ib-cr_aac(6')-Ib-cr10_aac(6')-Ib-cr11_aac(6')-Ib-cr4_aac(6')-Ib-cr5 | - |
| s_12849 | Quinolone | qnrS1 | - |
| s_13022 | Quinolone | - | - |
| s_13150 | Quinolone | qnrS1, aac(6')-Ib-D181Y_aac(6')-Ib-W104R_aac(6')-Ib-cr_aac(6')-Ib-cr10_aac(6')-Ib-cr11_aac(6')-Ib-cr4_aac(6')-Ib-cr5 | - |
| s_13959 | Quinolone | aac(6')-Ib-D181Y_aac(6')-Ib-W104R_aac(6')-Ib-cr_aac(6')-Ib-cr10_aac(6')-Ib-cr11_aac(6')-Ib-cr4_aac(6')-Ib-cr5 | - |
| s_13987 | Quinolone | aac(6')-Ib-D181Y_aac(6')-Ib-W104R_aac(6')-Ib-cr_aac(6')-Ib-cr10_aac(6')-Ib-cr11_aac(6')-Ib-cr4_aac(6')-Ib-cr5 | - |
| s_3117 | Quinolone | aac(6')-Ib-D181Y_aac(6')-Ib-W104R_aac(6')-Ib-cr_aac(6')-Ib-cr10_aac(6')-Ib-cr11_aac(6')-Ib-cr4_aac(6')-Ib-cr5 | - |
| s_316 | Quinolone | aac(6')-Ib-D181Y_aac(6')-Ib-W104R_aac(6')-Ib-cr_aac(6')-Ib-cr10_aac(6')-Ib-cr11_aac(6')-Ib-cr4_aac(6')-Ib-cr5, qepA8_qepA4 | - |
| s_317 | Quinolone | aac(6')-Ib-D181Y_aac(6')-Ib-W104R_aac(6')-Ib-cr_aac(6')-Ib-cr10_aac(6')-Ib-cr11_aac(6')-Ib-cr4_aac(6')-Ib-cr5 | - |
| s_4294 | Quinolone | qepA8_qepA4 | - |
| s_6558 | Quinolone | aac(6')-Ib-D181Y_aac(6')-Ib-W104R_aac(6')-Ib-cr_aac(6')-Ib-cr10_aac(6')-Ib-cr11_aac(6')-Ib-cr4_aac(6')-Ib-cr5 | - |
| s_90 | Quinolone | - | - |
| s_12116 | Streptomycin | aph(6)-Id, aadA5, aph(3'')-Ib | - |
| s_12117 | Streptomycin | aph(6)-Id, aadA5 | - |
| s_12155 | Streptomycin | aadA5 | - |
| s_12301 | Streptomycin | aadA5 | - |
| s_12414 | Streptomycin | aph(6)-Id, aph(3'')-Ib | - |
| s_12479 | Streptomycin | aph(6)-Id, aadA5, aph(3'')-Ib | - |
| s_12480 | Streptomycin | aph(6)-Id, aadA5, aph(3'')-Ib | - |
| s_12845 | Streptomycin | aph(6)-Id, aph(3'')-Ib | - |
| s_12849 | Streptomycin | - | - |
| s_13022 | Streptomycin | aph(6)-Id, aadA5, aph(3'')-Ib | - |
| s_13150 | Streptomycin | aph(6)-Id, aadA5, aph(3'')-Ib | - |
| s_13959 | Streptomycin | aph(6)-Id, aadA5, aph(3'')-Ib | - |
| s_13987 | Streptomycin | aph(6)-Id, aadA5, aph(3'')-Ib | - |
| s_3117 | Streptomycin | - | - |
| s_316 | Streptomycin | aph(6)-Id, aadA5, aph(3'')-Ib | - |
| s_317 | Streptomycin | - | - |
| s_4294 | Streptomycin | aadA2, aph(6)-Id, aph(3'')-Ib | - |
| s_6558 | Streptomycin | aph(6)-Id, aph(3'')-Ib | - |
| s_90 | Streptomycin | aph(6)-Id, aph(3'')-Ib | - |
| s_12116 | Sulfonamide | sul2, sul1 | - |
| s_12117 | Sulfonamide | sul1 | - |
| s_12155 | Sulfonamide | sul1 | - |
| s_12301 | Sulfonamide | sul2, sul1 | - |
| s_12414 | Sulfonamide | sul2 | - |
| s_12479 | Sulfonamide | sul2, sul1 | - |
| s_12480 | Sulfonamide | sul2, sul1 | - |
| s_12845 | Sulfonamide | sul2 | - |
| s_12849 | Sulfonamide | - | - |
| s_13022 | Sulfonamide | sul2, sul1 | - |
| s_13150 | Sulfonamide | sul2, sul1 | - |
| s_13959 | Sulfonamide | sul2, sul1 | - |
| s_13987 | Sulfonamide | sul2, sul1 | - |
| s_3117 | Sulfonamide | - | - |
| s_316 | Sulfonamide | sul2, sul1 | - |
| s_317 | Sulfonamide | - | - |
| s_4294 | Sulfonamide | sul2, sul1 | - |
| s_6558 | Sulfonamide | sul2 | - |
| s_90 | Sulfonamide | sul2 | - |
| s_12116 | Tetracycline | tet(B) | - |
| s_12117 | Tetracycline | tet(B) | - |
| s_12155 | Tetracycline | tet(B) | - |
| s_12301 | Tetracycline | tet(B) | - |
| s_12414 | Tetracycline | tet(A) | - |
| s_12479 | Tetracycline | tet(A) | - |
| s_12480 | Tetracycline | tet(B) | - |
| s_12845 | Tetracycline | tet(B) | - |
| s_12849 | Tetracycline | - | - |
| s_13022 | Tetracycline | tet(B) | - |
| s_13150 | Tetracycline | tet(B) | - |
| s_13959 | Tetracycline | tet(A) | - |
| s_13987 | Tetracycline | tet(A) | - |
| s_3117 | Tetracycline | tet(B) | - |
| s_316 | Tetracycline | tet(B) | - |
| s_317 | Tetracycline | tet(B) | - |
| s_4294 | Tetracycline | tet(B) | - |
| s_6558 | Tetracycline | tet(B) | - |
| s_90 | Tetracycline | tet(B) | - |
| s_12116 | Tobramycin | aac(6')-Ib-D181Y_aac(6')-Ib-W104R_aac(6')-Ib-cr_aac(6')-Ib-cr10_aac(6')-Ib-cr11_aac(6')-Ib-cr4_aac(6')-Ib-cr5 | - |
| s_12117 | Tobramycin | aac(6')-Ib-D181Y_aac(6')-Ib-W104R_aac(6')-Ib-cr_aac(6')-Ib-cr10_aac(6')-Ib-cr11_aac(6')-Ib-cr4_aac(6')-Ib-cr5 | - |
| s_12155 | Tobramycin | aac(6')-Ib-D181Y_aac(6')-Ib-W104R_aac(6')-Ib-cr_aac(6')-Ib-cr10_aac(6')-Ib-cr11_aac(6')-Ib-cr4_aac(6')-Ib-cr5 | - |
| s_12301 | Tobramycin | aac(6')-Ib-D181Y_aac(6')-Ib-W104R_aac(6')-Ib-cr_aac(6')-Ib-cr10_aac(6')-Ib-cr11_aac(6')-Ib-cr4_aac(6')-Ib-cr5 | - |
| s_12414 | Tobramycin | - | - |
| s_12479 | Tobramycin | aac(6')-Ib-D181Y_aac(6')-Ib-W104R_aac(6')-Ib-cr_aac(6')-Ib-cr10_aac(6')-Ib-cr11_aac(6')-Ib-cr4_aac(6')-Ib-cr5 | - |
| s_12480 | Tobramycin | aac(6')-Ib-D181Y_aac(6')-Ib-W104R_aac(6')-Ib-cr_aac(6')-Ib-cr10_aac(6')-Ib-cr11_aac(6')-Ib-cr4_aac(6')-Ib-cr5 | - |
| s_12845 | Tobramycin | aac(6')-Ib-D181Y_aac(6')-Ib-W104R_aac(6')-Ib-cr_aac(6')-Ib-cr10_aac(6')-Ib-cr11_aac(6')-Ib-cr4_aac(6')-Ib-cr5 | - |
| s_12849 | Tobramycin | - | - |
| s_13022 | Tobramycin | - | - |
| s_13150 | Tobramycin | aac(6')-Ib-D181Y_aac(6')-Ib-W104R_aac(6')-Ib-cr_aac(6')-Ib-cr10_aac(6')-Ib-cr11_aac(6')-Ib-cr4_aac(6')-Ib-cr5 | - |
| s_13959 | Tobramycin | aac(6')-Ib-D181Y_aac(6')-Ib-W104R_aac(6')-Ib-cr_aac(6')-Ib-cr10_aac(6')-Ib-cr11_aac(6')-Ib-cr4_aac(6')-Ib-cr5 | - |
| s_13987 | Tobramycin | aac(6')-Ib-D181Y_aac(6')-Ib-W104R_aac(6')-Ib-cr_aac(6')-Ib-cr10_aac(6')-Ib-cr11_aac(6')-Ib-cr4_aac(6')-Ib-cr5 | - |
| s_3117 | Tobramycin | aac(6')-Ib-D181Y_aac(6')-Ib-W104R_aac(6')-Ib-cr_aac(6')-Ib-cr10_aac(6')-Ib-cr11_aac(6')-Ib-cr4_aac(6')-Ib-cr5 | - |
| s_316 | Tobramycin | aac(6')-Ib-D181Y_aac(6')-Ib-W104R_aac(6')-Ib-cr_aac(6')-Ib-cr10_aac(6')-Ib-cr11_aac(6')-Ib-cr4_aac(6')-Ib-cr5 | - |
| s_317 | Tobramycin | aac(6')-Ib-D181Y_aac(6')-Ib-W104R_aac(6')-Ib-cr_aac(6')-Ib-cr10_aac(6')-Ib-cr11_aac(6')-Ib-cr4_aac(6')-Ib-cr5 | - |
| s_4294 | Tobramycin | - | - |
| s_6558 | Tobramycin | aac(6')-Ib-D181Y_aac(6')-Ib-W104R_aac(6')-Ib-cr_aac(6')-Ib-cr10_aac(6')-Ib-cr11_aac(6')-Ib-cr4_aac(6')-Ib-cr5 | - |
| s_90 | Tobramycin | - | - |
| s_12116 | Trimethoprim | dfrA17 | - |
| s_12117 | Trimethoprim | dfrA17 | - |
| s_12155 | Trimethoprim | dfrA17 | - |
| s_12301 | Trimethoprim | dfrA17 | - |
| s_12414 | Trimethoprim | dfrA14 | - |
| s_12479 | Trimethoprim | dfrA17 | - |
| s_12480 | Trimethoprim | dfrA17 | - |
| s_12845 | Trimethoprim | dfrA17 | - |
| s_12849 | Trimethoprim | - | - |
| s_13022 | Trimethoprim | dfrA17 | - |
| s_13150 | Trimethoprim | dfrA17 | - |
| s_13959 | Trimethoprim | dfrA17 | - |
| s_13987 | Trimethoprim | dfrA17 | - |
| s_3117 | Trimethoprim | - | - |
| s_316 | Trimethoprim | dfrA17 | - |
| s_317 | Trimethoprim | - | - |
| s_4294 | Trimethoprim | dfrA12 | - |
| s_6558 | Trimethoprim | dfrA17 | - |
| s_90 | Trimethoprim | dfrA8 | - |

The first column lists the sample name, the second column the name of the antibiotic or class of antibiotics. The third and fourth column contains the detected genes and mutations associated with resistance to the corresponding antibiotics, respectively. Note that a single gene or mutation can be associated with resistance to multiple antibiotics.

### **Table S7: Predicted genomic origin of detected AMR genes**

| **sample** | ***aac(3)-Ia*** | ***aac(3)-IId*** | ***aac(6')*** | ***aadA2*** | ***aadA5*** | ***aph(3'')-Ib*** | ***aph(6)-Id*** | ***blaCMY-42*** | ***blaCTX-M-15*** | ***blaEC-15*** | ***blaOXA-1*** | ***blaOXA-181*** | ***blaTEM-1*** | ***catA1*** | ***dfrA12*** | ***dfrA14*** | ***dfrA17*** | ***dfrA8*** | ***erm(B)*** | ***mph(A)*** | ***qepA4*** | ***qnrS1*** | ***sul1*** | ***sul2*** | ***tet(A)*** | ***tet(B)*** |
| --- | --- | --- | --- | --- | --- | --- | --- | --- | --- | --- | --- | --- | --- | --- | --- | --- | --- | --- | --- | --- | --- | --- | --- | --- | --- | --- |
| s_12116 | - | P | P | - | P | P | P | - | P | C | P | - | - | - | - | - | P | - | - | P | - | - | P | P | - | P |
| s_12117 | - | - | P | - | P | - | P | P | C | C | P | - | - | - | - | - | P | - | P | P | - | - | P | - | - | P |
| s_12155 | - | C | C | - | P | - | - | - | C | C | C | - | C | C | - | - | P | - | - | P | - | - | P | - | - | P |
| s_12301 | - | C | P | - | P | - | - | - | P | C | P | - | - | - | - | - | P | - | - | P | - | - | P | C | - | P |
| s_12414 | - | - | - | - | - | P | P | - | P | C | - | - | P | - | - | P | - | - | - | - | - | P | - | P | P | - |
| s_12479 | - | - | C | - | P | P | P | - | P | C | C | - | - | P | - | - | P | - | - | P | - | - | P | P | P | - |
| s_12480 | - | P | C | - | C | P | P | C | P | C | C | P | C | - | - | - | C | - | - | C | - | P | C | P | - | P |
| s_12845 | - | C | C | - | - | C | C | - | C | C | C | - | - | - | - | - | P | - | - | - | - | - | - | C | - | P |
| s_12849 | - | - | - | - | - | - | - | - | C | C | - | - | - | - | - | - | - | - | - | - | - | C | - | - | - | - |
| s_13022 | - | - | - | - | P | P | P | - | - | C | - | - | P | C | - | - | P | - | - | P | - | - | P | P | - | P |
| s_13150 | - | P | C | - | P | P | P | C | P | C | C | P | P | - | - | - | P | - | - | P | - | P | P | P | - | P |
| s_13959 | - | C | C | - | P | P | P | - | P | C | C | - | P | - | - | - | P | - | - | P | - | - | P | P | C | - |
| s_13987 | - | C | C | - | P | P | P | - | P | C | C | - | - | - | - | - | P | - | - | P | - | - | P | P | C | - |
| s_3117 | - | C | C | - | - | - | - | P | - | C | C | - | - | - | - | - | - | - | - | - | - | - | - | - | - | C |
| s_316 | C | P | C | - | P | P | P | - | P | C | C | - | P | - | - | - | P | - | - | - | P | - | P | P | - | P |
| s_317 | - | C | C | - | - | - | - | P | - | C | C | - | - | - | - | - | - | - | - | - | - | - | - | - | - | C |
| s_4294 | C | P | - | P | - | P | P | - | P | C | - | - | P | P | P | - | - | - | - | P | P | - | P | P | - | P |
| s_6558 | - | C | C | - | - | P | P | - | C | C | C | - | - | - | - | - | C | - | - | - | - | - | - | P | - | C |
| s_90 | - | - | - | - | - | P | P | - | - | C | - | - | P | - | - | - | - | P | - | - | - | - | - | P | - | P |

Abbreviations: Chromosome (C), plasmid (P). Genes that clustered together at 80% identity were merged into a single entry.

### **Table S8: Comparison predicted and observed AMR profiles.**

| **sample** | **Amikacin** | **Chloramphenicol** | **Ciprofloxacin** | **Gentamicin** | **Tobramycin** | **Trimethoprim** |
| --- | --- | --- | --- | --- | --- | --- |
| s_12116 | **FP** | TN | TP | TP | TP | TP |
| s_12117 | **FP** | TN | TP | **FN** | TP | TP |
| s_12155 | **FP** | TP | TP | TP | TP | TP |
| s_12301 | **FP** | TN | TP | TP | TP | TP |
| s_12414 | TN | TN | TN | TN | TN | TP |
| s_12479 | **FP** | TP | TP | TN | **FP** | TP |
| s_12480 | **FP** | TN | TP | TP | TP | TP |
| s_12845 | **FP** | TN | TP | TP | TP | TP |
| s_12849 | TN | TN | TN | TN | TN | TN |
| s_13022 | TN | TP | **FP** | TN | TN | TP |
| s_13150 | **FP** | TN | TP | TP | TP | TP |
| s_13959 | **FP** | TN | TP | TP | TP | TP |
| s_13987 | **FP** | TN | TP | TP | TP | TP |
| s_3117 | **FP** | TN | TP | TP | TP | TN |
| s_316 | **FP** | TN | TP | TP | TP | TP |
| s_317 | **FP** | TN | TP | TP | TP | TN |
| s_4294 | TN | TP | TP | TP | **FN** | **FP** |
| s_6558 | **FP** | TN | TP | TP | TP | TP |
| s_90 | TN | TN | **FP** | TN | TN | TP |

Corresponding definitions are provided in the Material and Methods. Mismatches are shown in yellow and indicated in bold. Abbreviations: True positive (TP); true negative (TN); false positive (FP); false negative (FN).

### **Table S9: SNP distance matrix for ST405**

|  | **ESC_CB2615AA_AS** | **ESC_EB5356AA_AS** | **ESC_FB3938AA_AS** | **ESC_LB0580AA_AS** | **ESC_LB2126AA** | **ESC_NB2585AA_AS** | **ESC_NB8273AA_AS** | **ESC_SA4848AA_AS** | **ESC_SA9385AA_AS** | **ESC_SA9400AA_AS** | **ESC_SA9436AA_AS** | **s_3117** | **s_317** |
| --- | --- | --- | --- | --- | --- | --- | --- | --- | --- | --- | --- | --- | --- |
| **ESC_CB2615AA_AS** | 0 | 6 | 4 | 6799 | 4110 | 4936 | 4 | 3547 | 10989 | 11085 | 10827 | 5397 | 5414 |
| **ESC_EB5356AA_AS** | 6 | 0 | 4 | 6739 | 3993 | 4920 | 4 | 3508 | 10955 | 11051 | 10791 | 5369 | 5384 |
| **ESC_FB3938AA_AS** | 4 | 4 | 0 | 6719 | 4072 | 4945 | 0 | 3543 | 10926 | 11022 | 10781 | 5373 | 5392 |
| **ESC_LB0580AA_AS** | 6799 | 6739 | 6719 | 0 | 9184 | 10584 | 6801 | 9253 | 9677 | 9749 | 9538 | 10939 | 10951 |
| **ESC_LB2126AA** | 4110 | 3993 | 4072 | 9184 | 0 | 6168 | 4150 | 4300 | 13638 | 13822 | 13418 | 6722 | 6734 |
| **ESC_NB2585AA_AS** | 4936 | 4920 | 4945 | 10584 | 6168 | 0 | 4963 | 3145 | 14871 | 14990 | 14654 | 725 | 734 |
| **ESC_NB8273AA_AS** | 4 | 4 | 0 | 6801 | 4150 | 4963 | 0 | 3568 | 10997 | 11097 | 10839 | 5422 | 5436 |
| **ESC_SA4848AA_AS** | 3547 | 3508 | 3543 | 9253 | 4300 | 3145 | 3568 | 0 | 13321 | 13465 | 13083 | 3915 | 3931 |
| **ESC_SA9385AA_AS** | 10989 | 10955 | 10926 | 9677 | 13638 | 14871 | 10997 | 13321 | 0 | 4 | 74 | 14974 | 14988 |
| **ESC_SA9400AA_AS** | 11085 | 11051 | 11022 | 9749 | 13822 | 14990 | 11097 | 13465 | 4 | 0 | 71 | 15089 | 15104 |
| **ESC_SA9436AA_AS** | 10827 | 10791 | 10781 | 9538 | 13418 | 14654 | 10839 | 13083 | 74 | 71 | 0 | 14772 | 14786 |
| **s_3117** | 5397 | 5369 | 5373 | 10939 | 6722 | 725 | 5422 | 3915 | 14974 | 15089 | 14772 | 0 | 0 |
| **s_317** | 5414 | 5384 | 5392 | 10951 | 6734 | 734 | 5436 | 3931 | 14988 | 15104 | 14786 | 0 | 0 |

### **Table S10: SNP distance matrix for ST131**

|  | ESC_DB0443AA_AS | ESC_EB4935AA_AS | ESC_EB5141AA_AS | ESC_FB6767AA_AS | ESC_GB9896AA_AS | ESC_HB0221AA_AS | ESC_IB6568AA_AS | ESC_IB6569AA_AS | ESC_IB6570AA_AS | ESC_IB6574AA_AS | ESC_IB6575AA_AS | ESC_IB6576AA_AS | ESC_IB6577AA_AS | ESC_IB6578AA_AS | ESC_IB6579AA_AS | ESC_IB6580AA_AS | ESC_IB6581AA_AS | ESC_IB6583AA_AS | ESC_JB0080AA_AS | ESC_JB2945AA_AS | ESC_JB5273AA_AS | ESC_JB8801AA_AS | ESC_JB8945AA_AS | ESC_JB9397AA_AS | ESC_LB2114AA | ESC_LB8006AA_AS | ESC_MB8658AA_AS | ESC_NB7876AA_AS | ESC_NB8772AA_AS | ESC_NB9946AA_AS | ESC_SA1429AA_AS | ESC_SA2416AA_AS | ESC_SA4772AA_AS | ESC_TA7442AA_AS | ESC_TA8034AA_AS | ESC_TA8124AA_AS | ESC_TA8164AA_AS | ESC_TA8186AA_AS | ESC_UA2586AA_AS | ESC_UA2801AA_AS | ESC_UA3662AA_AS | ESC_ZA2787AA_AS | s_13959 | s_13987_1 |
| --- | --- | --- | --- | --- | --- | --- | --- | --- | --- | --- | --- | --- | --- | --- | --- | --- | --- | --- | --- | --- | --- | --- | --- | --- | --- | --- | --- | --- | --- | --- | --- | --- | --- | --- | --- | --- | --- | --- | --- | --- | --- | --- | --- | --- |
| ESC_DB0443AA_AS | 0 | 1655 | 1611 | 1408 | 7486 | 1166 | 1356 | 7477 | 14757 | 8125 | 7643 | 7341 | 1356 | 7544 | 14888 | 7627 | 7069 | 7883 | 14162 | 15254 | 6814 | 14437 | 1163 | 14292 | 2303 | 2099 | 6375 | 13572 | 1795 | 7703 | 13970 | 1355 | 6567 | 1603 | 6402 | 15174 | 6388 | 1613 | 14659 | 14645 | 1458 | 1348 | 1546 | 1611 |
| ESC_EB4935AA_AS | 1655 | 0 | 944 | 198 | 7233 | 472 | 158 | 7649 | 14597 | 8018 | 7837 | 8091 | 159 | 7712 | 14706 | 7777 | 7774 | 7748 | 14688 | 15339 | 7752 | 14262 | 468 | 14103 | 1198 | 1011 | 6139 | 12987 | 1054 | 7668 | 13971 | 137 | 6314 | 812 | 6405 | 15135 | 6380 | 840 | 14476 | 14468 | 266 | 140 | 1043 | 961 |
| ESC_EB5141AA_AS | 1611 | 944 | 0 | 988 | 7823 | 720 | 951 | 8187 | 14382 | 8573 | 8387 | 7921 | 1005 | 8292 | 14548 | 8295 | 7581 | 8280 | 14699 | 15303 | 7224 | 14128 | 719 | 13962 | 1637 | 1265 | 6694 | 12997 | 1427 | 8022 | 13834 | 942 | 6858 | 1581 | 6726 | 15049 | 6698 | 1609 | 14488 | 14484 | 1062 | 937 | 1585 | 1656 |
| ESC_FB6767AA_AS | 1408 | 198 | 988 | 0 | 6201 | 439 | 130 | 7358 | 14317 | 7811 | 7571 | 7661 | 130 | 7402 | 14426 | 7546 | 7405 | 7580 | 14033 | 14225 | 6939 | 14087 | 421 | 14026 | 879 | 844 | 5921 | 12616 | 876 | 7462 | 13891 | 182 | 6224 | 116 | 6190 | 13935 | 6177 | 116 | 14189 | 14194 | 149 | 125 | 329 | 191 |
| ESC_GB9896AA_AS | 7486 | 7233 | 7823 | 6201 | 0 | 7158 | 7135 | 3073 | 13658 | 2760 | 2902 | 6510 | 7139 | 3093 | 13723 | 2886 | 6326 | 2619 | 13578 | 14511 | 8167 | 13807 | 7162 | 13535 | 8569 | 8099 | 82 | 13022 | 8227 | 1850 | 13393 | 7108 | 112 | 6305 | 355 | 14129 | 347 | 6318 | 13743 | 13745 | 7056 | 6411 | 7461 | 7337 |
| ESC_HB0221AA_AS | 1166 | 472 | 720 | 439 | 7158 | 0 | 386 | 7216 | 13534 | 7439 | 7282 | 6693 | 387 | 7214 | 13619 | 7280 | 6488 | 7334 | 13388 | 14147 | 6467 | 13413 | 0 | 13276 | 885 | 848 | 5964 | 11960 | 678 | 7365 | 13235 | 465 | 6234 | 435 | 5987 | 13927 | 5964 | 434 | 13545 | 13548 | 384 | 372 | 489 | 484 |
| ESC_IB6568AA_AS | 1356 | 158 | 951 | 130 | 7135 | 386 | 0 | 7335 | 14232 | 7783 | 7516 | 7642 | 0 | 7364 | 14343 | 7498 | 7377 | 7548 | 13956 | 14804 | 6971 | 13995 | 369 | 13979 | 837 | 806 | 5910 | 12623 | 827 | 7445 | 13835 | 124 | 6228 | 124 | 6203 | 14529 | 6189 | 125 | 14180 | 14194 | 86 | 70 | 291 | 143 |
| ESC_IB6569AA_AS | 7477 | 7649 | 8187 | 7358 | 3073 | 7216 | 7335 | 0 | 14841 | 931 | 412 | 4254 | 7338 | 0 | 14950 | 403 | 4088 | 809 | 14620 | 15072 | 8142 | 14872 | 7219 | 14549 | 9043 | 8414 | 2850 | 14004 | 8505 | 4579 | 14437 | 7304 | 3120 | 7533 | 3198 | 14652 | 3187 | 7538 | 15055 | 15044 | 7253 | 7290 | 7720 | 7432 |
| ESC_IB6570AA_AS | 14757 | 14597 | 14382 | 14317 | 13658 | 13534 | 14232 | 14841 | 0 | 14805 | 14843 | 14881 | 14231 | 14408 | 0 | 14852 | 14757 | 14662 | 510 | 493 | 16647 | 142 | 13543 | 431 | 15336 | 14817 | 13371 | 1787 | 14988 | 13592 | 411 | 14420 | 13755 | 14361 | 13383 | 414 | 13381 | 14348 | 603 | 604 | 14249 | 14257 | 14245 | 14126 |
| ESC_IB6574AA_AS | 8125 | 8018 | 8573 | 7811 | 2760 | 7439 | 7783 | 931 | 14805 | 0 | 395 | 3979 | 7746 | 892 | 14862 | 468 | 3915 | 0 | 14959 | 15162 | 8347 | 15206 | 7439 | 14678 | 9659 | 9001 | 2763 | 14156 | 9055 | 4488 | 14811 | 7792 | 2748 | 7944 | 3124 | 14735 | 3129 | 7949 | 15047 | 15063 | 7734 | 7755 | 8101 | 7823 |
| ESC_IB6575AA_AS | 7643 | 7837 | 8387 | 7571 | 2902 | 7282 | 7516 | 412 | 14843 | 395 | 0 | 4037 | 7519 | 428 | 14947 | 0 | 3952 | 345 | 14684 | 15122 | 8184 | 15016 | 7283 | 14670 | 9341 | 8759 | 2940 | 14094 | 8736 | 4662 | 14496 | 7533 | 2953 | 7720 | 3286 | 14716 | 3272 | 7734 | 15079 | 15082 | 7469 | 7521 | 7921 | 7647 |
| ESC_IB6576AA_AS | 7341 | 8091 | 7921 | 7661 | 6510 | 6693 | 7642 | 4254 | 14881 | 3979 | 4037 | 0 | 7612 | 4198 | 14944 | 4106 | 0 | 3888 | 14837 | 14725 | 7425 | 14187 | 6689 | 14147 | 9496 | 8625 | 6427 | 14155 | 8869 | 7953 | 14457 | 7690 | 6529 | 7863 | 6599 | 14839 | 6597 | 7860 | 14057 | 14075 | 7810 | 7621 | 8003 | 7872 |
| ESC_IB6577AA_AS | 1356 | 159 | 1005 | 130 | 7139 | 387 | 0 | 7338 | 14231 | 7746 | 7519 | 7612 | 0 | 7367 | 14342 | 7501 | 7384 | 7549 | 13958 | 14809 | 6972 | 13998 | 370 | 13978 | 895 | 867 | 5914 | 12625 | 833 | 7450 | 13834 | 160 | 6233 | 124 | 6207 | 14532 | 6193 | 129 | 14188 | 14202 | 104 | 70 | 344 | 196 |
| ESC_IB6578AA_AS | 7544 | 7712 | 8292 | 7402 | 3093 | 7214 | 7364 | 0 | 14408 | 892 | 428 | 4198 | 7367 | 0 | 14502 | 418 | 4100 | 820 | 14313 | 14728 | 8203 | 14522 | 7216 | 14170 | 9194 | 8528 | 2855 | 13635 | 8608 | 4573 | 14114 | 7403 | 3134 | 7593 | 3220 | 14276 | 3208 | 7603 | 14756 | 14763 | 7330 | 7336 | 7796 | 7519 |
| ESC_IB6579AA_AS | 14888 | 14706 | 14548 | 14426 | 13723 | 13619 | 14343 | 14950 | 0 | 14862 | 14947 | 14944 | 14342 | 14502 | 0 | 14948 | 14823 | 14747 | 532 | 511 | 16744 | 150 | 13626 | 452 | 15557 | 15010 | 13425 | 1796 | 15128 | 13609 | 437 | 14541 | 13820 | 14493 | 13447 | 438 | 13444 | 14485 | 625 | 627 | 14352 | 14357 | 14420 | 14312 |
| ESC_IB6580AA_AS | 7627 | 7777 | 8295 | 7546 | 2886 | 7280 | 7498 | 403 | 14852 | 468 | 0 | 4106 | 7501 | 418 | 14948 | 0 | 3946 | 351 | 14666 | 15120 | 8157 | 15014 | 7282 | 14667 | 9205 | 8639 | 2925 | 14085 | 8678 | 4647 | 14480 | 7478 | 2938 | 7687 | 3264 | 14675 | 3260 | 7697 | 15054 | 15067 | 7432 | 7495 | 7832 | 7553 |
| ESC_IB6581AA_AS | 7069 | 7774 | 7581 | 7405 | 6326 | 6488 | 7377 | 4088 | 14757 | 3915 | 3952 | 0 | 7384 | 4100 | 14823 | 3946 | 0 | 3825 | 14634 | 14537 | 7222 | 14034 | 6484 | 14020 | 9063 | 8219 | 6245 | 13971 | 8546 | 7760 | 14285 | 7388 | 6338 | 7585 | 6421 | 14669 | 6412 | 7574 | 13899 | 13912 | 7547 | 7360 | 7658 | 7536 |
| ESC_IB6583AA_AS | 7883 | 7748 | 8280 | 7580 | 2619 | 7334 | 7548 | 809 | 14662 | 0 | 345 | 3888 | 7549 | 820 | 14747 | 351 | 3825 | 0 | 14758 | 14984 | 8129 | 15047 | 7332 | 14534 | 9285 | 8649 | 2618 | 14012 | 8764 | 4347 | 14628 | 7531 | 2606 | 7690 | 2978 | 14569 | 2971 | 7691 | 14878 | 14900 | 7483 | 7520 | 7810 | 7526 |
| ESC_JB0080AA_AS | 14162 | 14688 | 14699 | 14033 | 13578 | 13388 | 13956 | 14620 | 510 | 14959 | 14684 | 14837 | 13958 | 14313 | 532 | 14666 | 14634 | 14758 | 0 | 531 | 16483 | 569 | 13388 | 624 | 15903 | 15232 | 13296 | 1816 | 15017 | 13591 | 182 | 13892 | 13658 | 14529 | 13500 | 1140 | 13487 | 14542 | 973 | 960 | 13936 | 13979 | 13998 | 13887 |
| ESC_JB2945AA_AS | 15254 | 15339 | 15303 | 14225 | 14511 | 14147 | 14804 | 15072 | 493 | 15162 | 15122 | 14725 | 14809 | 14728 | 511 | 15120 | 14537 | 14984 | 531 | 0 | 16596 | 563 | 14149 | 567 | 16147 | 15677 | 13509 | 1779 | 15458 | 13787 | 224 | 14837 | 13911 | 14605 | 13523 | 1213 | 13516 | 14607 | 948 | 949 | 14785 | 14340 | 15150 | 15037 |
| ESC_JB5273AA_AS | 6814 | 7752 | 7224 | 6939 | 8167 | 6467 | 6971 | 8142 | 16647 | 8347 | 8184 | 7425 | 6972 | 8203 | 16744 | 8157 | 7222 | 8129 | 16483 | 16596 | 0 | 16442 | 6436 | 15604 | 8176 | 7780 | 8067 | 15907 | 7161 | 9650 | 15952 | 6988 | 8254 | 7277 | 8036 | 16733 | 8024 | 7284 | 16092 | 16052 | 7119 | 6903 | 7428 | 7288 |
| ESC_JB8801AA_AS | 14437 | 14262 | 14128 | 14087 | 13807 | 13413 | 13995 | 14872 | 142 | 15206 | 15016 | 14187 | 13998 | 14522 | 150 | 15014 | 14034 | 15047 | 569 | 563 | 16442 | 0 | 13417 | 517 | 14995 | 14484 | 13462 | 1718 | 14668 | 13744 | 497 | 14064 | 13890 | 14177 | 13477 | 563 | 13467 | 14179 | 1027 | 1029 | 13950 | 14039 | 14040 | 13960 |
| ESC_JB8945AA_AS | 1163 | 468 | 719 | 421 | 7162 | 0 | 369 | 7219 | 13543 | 7439 | 7283 | 6689 | 370 | 7216 | 13626 | 7282 | 6484 | 7332 | 13388 | 14149 | 6436 | 13417 | 0 | 13275 | 850 | 812 | 5965 | 11960 | 663 | 7368 | 13238 | 457 | 6246 | 416 | 5989 | 13916 | 5968 | 416 | 13547 | 13551 | 365 | 355 | 471 | 466 |
| ESC_JB9397AA_AS | 14292 | 14103 | 13962 | 14026 | 13535 | 13276 | 13979 | 14549 | 431 | 14678 | 14670 | 14147 | 13978 | 14170 | 452 | 14667 | 14020 | 14534 | 624 | 567 | 15604 | 517 | 13275 | 0 | 15117 | 14515 | 13161 | 1788 | 14607 | 13436 | 237 | 13871 | 13582 | 14114 | 13256 | 473 | 13244 | 14119 | 558 | 562 | 13901 | 13972 | 13993 | 13905 |
| ESC_LB2114AA | 2303 | 1198 | 1637 | 879 | 8569 | 885 | 837 | 9043 | 15336 | 9659 | 9341 | 9496 | 895 | 9194 | 15557 | 9205 | 9063 | 9285 | 15903 | 16147 | 8176 | 14995 | 850 | 15117 | 0 | 373 | 6860 | 13983 | 181 | 8121 | 14903 | 1161 | 7637 | 1560 | 7223 | 15882 | 7183 | 1586 | 15122 | 15109 | 1048 | 827 | 1707 | 1655 |
| ESC_LB8006AA_AS | 2099 | 1011 | 1265 | 844 | 8099 | 848 | 806 | 8414 | 14817 | 9001 | 8759 | 8625 | 867 | 8528 | 15010 | 8639 | 8219 | 8649 | 15232 | 15677 | 7780 | 14484 | 812 | 14515 | 373 | 0 | 6641 | 13458 | 401 | 7911 | 14398 | 987 | 7174 | 1487 | 6749 | 15377 | 6710 | 1517 | 14440 | 14442 | 848 | 809 | 1674 | 1611 |
| ESC_MB8658AA_AS | 6375 | 6139 | 6694 | 5921 | 82 | 5964 | 5910 | 2850 | 13371 | 2763 | 2940 | 6427 | 5914 | 2855 | 13425 | 2925 | 6245 | 2618 | 13296 | 13509 | 8067 | 13462 | 5965 | 13161 | 6860 | 6641 | 0 | 12635 | 6661 | 1844 | 13178 | 6017 | 111 | 6001 | 320 | 13111 | 316 | 6007 | 13528 | 13535 | 5835 | 5911 | 6217 | 6090 |
| ESC_NB7876AA_AS | 13572 | 12987 | 12997 | 12616 | 13022 | 11960 | 12623 | 14004 | 1787 | 14156 | 14094 | 14155 | 12625 | 13635 | 1796 | 14085 | 13971 | 14012 | 1816 | 1779 | 15907 | 1718 | 11960 | 1788 | 13983 | 13458 | 12635 | 0 | 13619 | 13043 | 1758 | 12756 | 13024 | 12653 | 12660 | 1704 | 12645 | 12651 | 2041 | 2045 | 12605 | 12626 | 12720 | 12733 |
| ESC_NB8772AA_AS | 1795 | 1054 | 1427 | 876 | 8227 | 678 | 827 | 8505 | 14988 | 9055 | 8736 | 8869 | 833 | 8608 | 15128 | 8678 | 8546 | 8764 | 15017 | 15458 | 7161 | 14668 | 663 | 14607 | 181 | 401 | 6661 | 13619 | 0 | 7924 | 14463 | 990 | 7320 | 1150 | 6970 | 15381 | 6947 | 1158 | 14828 | 14829 | 1044 | 808 | 1184 | 1131 |
| ESC_NB9946AA_AS | 7703 | 7668 | 8022 | 7462 | 1850 | 7365 | 7445 | 4579 | 13592 | 4488 | 4662 | 7953 | 7450 | 4573 | 13609 | 4647 | 7760 | 4347 | 13591 | 13787 | 9650 | 13744 | 7368 | 13436 | 8121 | 7911 | 1844 | 13043 | 7924 | 0 | 13484 | 7554 | 1864 | 7551 | 1987 | 13392 | 1982 | 7555 | 13815 | 13831 | 7373 | 7446 | 7494 | 7633 |
| ESC_SA1429AA_AS | 13970 | 13971 | 13834 | 13891 | 13393 | 13235 | 13835 | 14437 | 411 | 14811 | 14496 | 14457 | 13834 | 14114 | 437 | 14480 | 14285 | 14628 | 182 | 224 | 15952 | 497 | 13238 | 237 | 14903 | 14398 | 13178 | 1758 | 14463 | 13484 | 0 | 13710 | 13467 | 14008 | 13287 | 575 | 13271 | 14013 | 903 | 910 | 13781 | 13832 | 13924 | 13823 |
| ESC_SA2416AA_AS | 1355 | 137 | 942 | 182 | 7108 | 465 | 124 | 7304 | 14420 | 7792 | 7533 | 7690 | 160 | 7403 | 14541 | 7478 | 7388 | 7531 | 13892 | 14837 | 6988 | 14064 | 457 | 13871 | 1161 | 987 | 6017 | 12756 | 990 | 7554 | 13710 | 0 | 6195 | 178 | 6271 | 14702 | 6257 | 182 | 14267 | 14261 | 255 | 122 | 294 | 149 |
| ESC_SA4772AA_AS | 6567 | 6314 | 6858 | 6224 | 112 | 6234 | 6228 | 3120 | 13755 | 2748 | 2953 | 6529 | 6233 | 3134 | 13820 | 2938 | 6338 | 2606 | 13658 | 13911 | 8254 | 13890 | 6246 | 13582 | 7637 | 7174 | 111 | 13024 | 7320 | 1864 | 13467 | 6195 | 0 | 6320 | 378 | 13559 | 370 | 6319 | 13838 | 13849 | 6167 | 6221 | 6542 | 6412 |
| ESC_TA7442AA_AS | 1603 | 812 | 1581 | 116 | 6305 | 435 | 124 | 7533 | 14361 | 7944 | 7720 | 7863 | 124 | 7593 | 14493 | 7687 | 7585 | 7690 | 14529 | 14605 | 7277 | 14177 | 416 | 14114 | 1560 | 1487 | 6001 | 12653 | 1150 | 7551 | 14008 | 178 | 6320 | 0 | 6297 | 14358 | 6271 | 1 | 14320 | 14320 | 158 | 120 | 663 | 560 |
| ESC_TA8034AA_AS | 6402 | 6405 | 6726 | 6190 | 355 | 5987 | 6203 | 3198 | 13383 | 3124 | 3286 | 6599 | 6207 | 3220 | 13447 | 3264 | 6421 | 2978 | 13500 | 13523 | 8036 | 13477 | 5989 | 13256 | 7223 | 6749 | 320 | 12660 | 6970 | 1987 | 13287 | 6271 | 378 | 6297 | 0 | 13151 | 0 | 6300 | 13617 | 13616 | 6114 | 6192 | 6431 | 6376 |
| ESC_TA8124AA_AS | 15174 | 15135 | 15049 | 13935 | 14129 | 13927 | 14529 | 14652 | 414 | 14735 | 14716 | 14839 | 14532 | 14276 | 438 | 14675 | 14669 | 14569 | 1140 | 1213 | 16733 | 563 | 13916 | 473 | 15882 | 15377 | 13111 | 1704 | 15381 | 13392 | 575 | 14702 | 13559 | 14358 | 13151 | 0 | 13133 | 14373 | 722 | 726 | 14476 | 14045 | 15170 | 15111 |
| ESC_TA8164AA_AS | 6388 | 6380 | 6698 | 6177 | 347 | 5964 | 6189 | 3187 | 13381 | 3129 | 3272 | 6597 | 6193 | 3208 | 13444 | 3260 | 6412 | 2971 | 13487 | 13516 | 8024 | 13467 | 5968 | 13244 | 7183 | 6710 | 316 | 12645 | 6947 | 1982 | 13271 | 6257 | 370 | 6271 | 0 | 13133 | 0 | 6275 | 13601 | 13604 | 6110 | 6176 | 6417 | 6357 |
| ESC_TA8186AA_AS | 1613 | 840 | 1609 | 116 | 6318 | 434 | 125 | 7538 | 14348 | 7949 | 7734 | 7860 | 129 | 7603 | 14485 | 7697 | 7574 | 7691 | 14542 | 14607 | 7284 | 14179 | 416 | 14119 | 1586 | 1517 | 6007 | 12651 | 1158 | 7555 | 14013 | 182 | 6319 | 1 | 6300 | 14373 | 6275 | 0 | 14326 | 14318 | 159 | 121 | 679 | 580 |
| ESC_UA2586AA_AS | 14659 | 14476 | 14488 | 14189 | 13743 | 13545 | 14180 | 15055 | 603 | 15047 | 15079 | 14057 | 14188 | 14756 | 625 | 15054 | 13899 | 14878 | 973 | 948 | 16092 | 1027 | 13547 | 558 | 15122 | 14440 | 13528 | 2041 | 14828 | 13815 | 903 | 14267 | 13838 | 14320 | 13617 | 722 | 13601 | 14326 | 0 | 28 | 14161 | 14133 | 14325 | 14191 |
| ESC_UA2801AA_AS | 14645 | 14468 | 14484 | 14194 | 13745 | 13548 | 14194 | 15044 | 604 | 15063 | 15082 | 14075 | 14202 | 14763 | 627 | 15067 | 13912 | 14900 | 960 | 949 | 16052 | 1029 | 13551 | 562 | 15109 | 14442 | 13535 | 2045 | 14829 | 13831 | 910 | 14261 | 13849 | 14320 | 13616 | 726 | 13604 | 14318 | 28 | 0 | 14170 | 14137 | 14327 | 14191 |
| ESC_UA3662AA_AS | 1458 | 266 | 1062 | 149 | 7056 | 384 | 86 | 7253 | 14249 | 7734 | 7469 | 7810 | 104 | 7330 | 14352 | 7432 | 7547 | 7483 | 13936 | 14785 | 7119 | 13950 | 365 | 13901 | 1048 | 848 | 5835 | 12605 | 1044 | 7373 | 13781 | 255 | 6167 | 158 | 6114 | 14476 | 6110 | 159 | 14161 | 14170 | 0 | 76 | 308 | 178 |
| ESC_ZA2787AA_AS | 1348 | 140 | 937 | 125 | 6411 | 372 | 70 | 7290 | 14257 | 7755 | 7521 | 7621 | 70 | 7336 | 14357 | 7495 | 7360 | 7520 | 13979 | 14340 | 6903 | 14039 | 355 | 13972 | 827 | 809 | 5911 | 12626 | 808 | 7446 | 13832 | 122 | 6221 | 120 | 6192 | 14045 | 6176 | 121 | 14133 | 14137 | 76 | 0 | 279 | 135 |
| s_13959 | 1546 | 1043 | 1585 | 329 | 7461 | 489 | 291 | 7720 | 14245 | 8101 | 7921 | 8003 | 344 | 7796 | 14420 | 7832 | 7658 | 7810 | 13998 | 15150 | 7428 | 14040 | 471 | 13993 | 1707 | 1674 | 6217 | 12720 | 1184 | 7494 | 13924 | 294 | 6542 | 663 | 6431 | 15170 | 6417 | 679 | 14325 | 14327 | 308 | 279 | 0 | 201 |
| s_13987 | 1611 | 961 | 1656 | 191 | 7337 | 484 | 143 | 7432 | 14126 | 7823 | 7647 | 7872 | 196 | 7519 | 14312 | 7553 | 7536 | 7526 | 13887 | 15037 | 7288 | 13960 | 466 | 13905 | 1655 | 1611 | 6090 | 12733 | 1131 | 7633 | 13823 | 149 | 6412 | 560 | 6376 | 15111 | 6357 | 580 | 14191 | 14191 | 178 | 135 | 201 | 0 |

### **Table S11: SNP distance matrix for ST1193**

|  | **ESC_FB9069AA_AS** | **ESC_LB3394AA_AS** | **ESC_MB1813AA_AS** | **ESC_WA1759AA** | **s_12845** | **s_6558** |
| --- | --- | --- | --- | --- | --- | --- |
| **ESC_FB9069AA_AS** | 0 | 236 | 302 | 119 | 67 | 354 |
| **ESC_LB3394AA_AS** | 236 | 0 | 492 | 603 | 282 | 542 |
| **ESC_MB1813AA_AS** | 302 | 492 | 0 | 320 | 241 | 314 |
| **ESC_WA1759AA** | 119 | 603 | 320 | 0 | 121 | 433 |
| **s_12845** | 67 | 282 | 241 | 121 | 0 | 288 |
| **s_6558** | 354 | 542 | 314 | 433 | 288 | 0 |

### **Table S12: SNP distance matrix for ST410**

|  | **ESC_GA4687AA** | **ESC_FB9431AA_AS** | **ESC_LB5909AA_AS** | **ESC_LB5910AA_AS** | **ESC_LB5918AA_AS** | **ESC_UB1905AA_AS** | **ESC_NB7682AA_AS** | **ESC_LB2143AA_AS** | **ESC_LB5911AA_AS** | **s_12480** | **s_13150** | **ESC_LB5912AA_AS** |
| --- | --- | --- | --- | --- | --- | --- | --- | --- | --- | --- | --- | --- |
| **ESC_GA4687AA** | 0 | 4203 | 4181 | 4299 | 3699 | 17964 | 5489 | 39041 | 4303 | 4493 | 4457 | 4351 |
| **ESC_FB9431AA_AS** | 4203 | 0 | 455 | 459 | 1501 | 17538 | 5318 | 38322 | 459 | 464 | 460 | 463 |
| **ESC_LB5909AA_AS** | 4181 | 455 | 0 | 0 | 1549 | 16900 | 5188 | 37541 | 2 | 73 | 66 | 5 |
| **ESC_LB5910AA_AS** | 4299 | 459 | 0 | 0 | 1580 | 17213 | 5251 | 37959 | 3 | 68 | 61 | 5 |
| **ESC_LB5918AA_AS** | 3699 | 1501 | 1549 | 1580 | 0 | 17935 | 6506 | 38938 | 1590 | 1607 | 1618 | 1600 |
| **ESC_UB1905AA_AS** | 17964 | 17538 | 16900 | 17213 | 17935 | 0 | 14192 | 38873 | 17152 | 17456 | 17784 | 17236 |
| **ESC_NB7682AA_AS** | 5489 | 5318 | 5188 | 5251 | 6506 | 14192 | 0 | 36595 | 5257 | 5343 | 5609 | 5254 |
| **ESC_LB2143AA_AS** | 39041 | 38322 | 37541 | 37959 | 38938 | 38873 | 36595 | 0 | 37929 | 38306 | 38643 | 38047 |
| **ESC_LB5911AA_AS** | 4303 | 459 | 2 | 3 | 1590 | 17152 | 5257 | 37929 | 0 | 75 | 69 | 8 |
| **s_12480** | 4493 | 464 | 73 | 68 | 1607 | 17456 | 5343 | 38306 | 75 | 0 | 70 | 82 |
| **s_13150** | 4457 | 460 | 66 | 61 | 1618 | 17784 | 5609 | 38643 | 69 | 70 | 0 | 76 |
| **ESC_LB5912AA_AS** | 4351 | 463 | 5 | 5 | 1600 | 17236 | 5254 | 38047 | 8 | 82 | 76 | 0 |

## **Supplementary Figures**

### **Figure S1: Minimum spanning trees containing results of SNP typing for the samples classified as ST38 and ST617.**

| 1. **ST38** |
| --- |
| **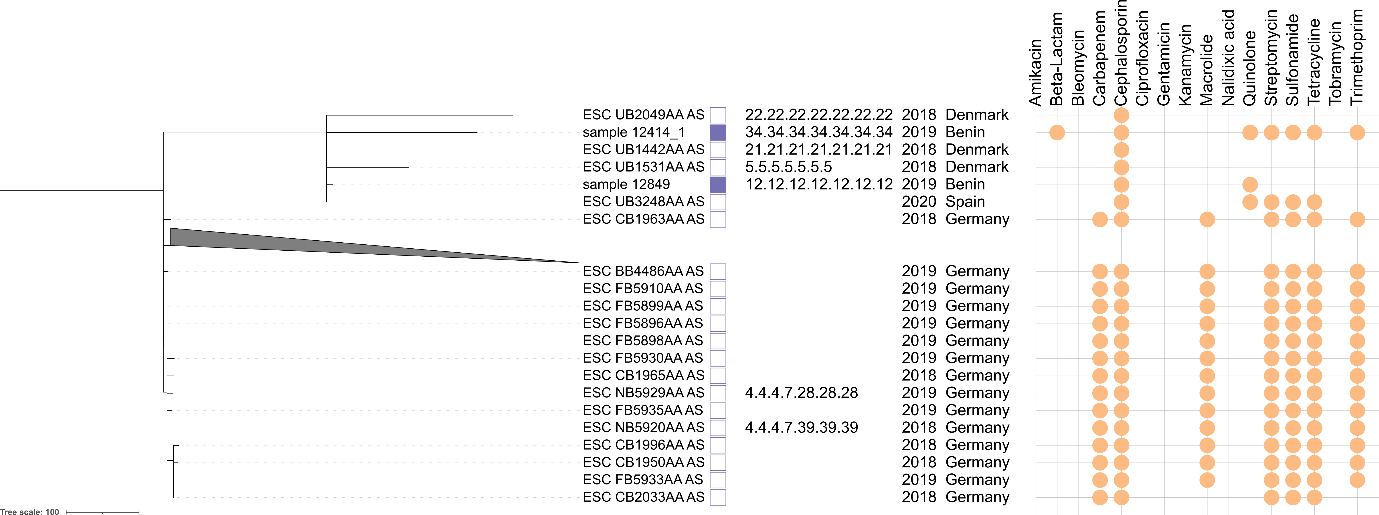** |
| 1. **ST617** |
| 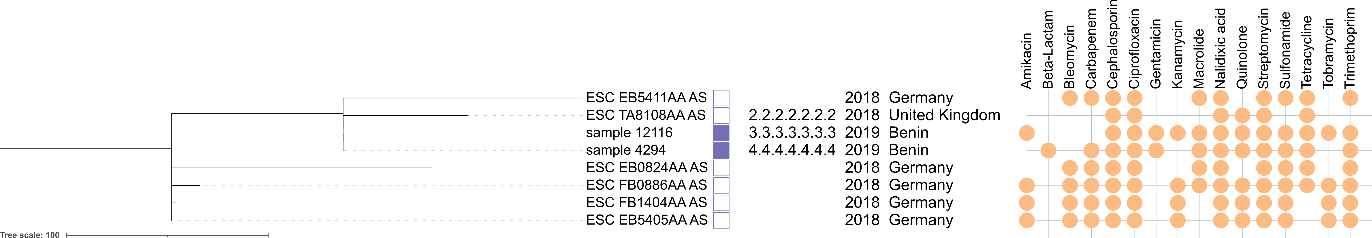 |

MSTs based on cgMLST results. The scale bar is expressed as number of allele differences. Annotations are (from left to right): sample name, sample origin (this study with a filled blue box or EnteroBase with an empty box), SNP address, isolation year, isolation country, and predicted AMR susceptibility. Samples with missing SNP addresses did not have publicly available Illumina paired-end data or were filtered out because their read coverage was too low. Predicted AMR susceptibility is shown for the 16 antibiotics that were most predicted (full results are provided in Table S6). In subfigure A the triangle represents 120 nodes that were collapsed for visual clarity.

### **Figure S2: Explanation of the SNP address for ST410**


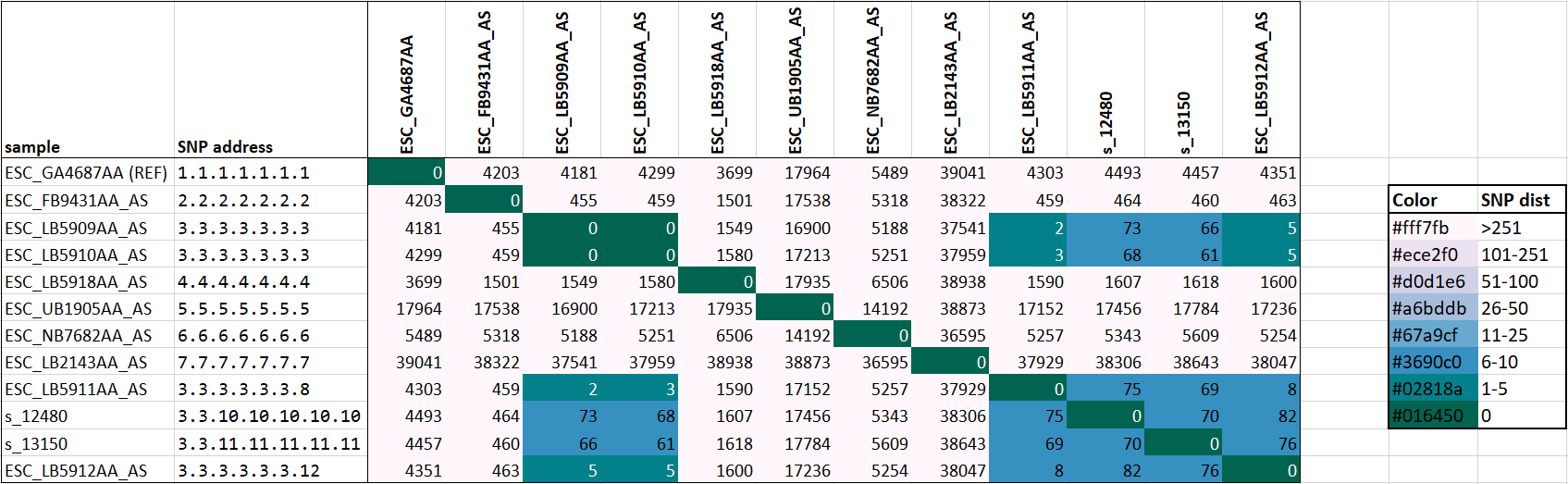


This figure represents the distance matrix for the phylogeny of ST410 (Figure 3d). SNP addresses for the other clusters were determined using the same methodology. Cells are colored according to the number of SNP differences following the threshold values used by the SNP address methodology as indicated on the right. The distance matrix contains the pairwise SNP distances that were used to construct the SNP addresses (obtained using the PHEnix and SnapperDB software packages). These pairwise SNP distances are calculated on shared high-quality positions, which can be different for each pair-wise comparison. Based on this distance matrix the SNP addresses were obtained as follows:

- The reference genome (ESC_GA4687AA) is, by convention, inserted in the database, corresponding to SNP address 1.1.1.1.1.1.1.
- Sample ESC_FB9431AA_AS is inserted into the database, and since this sample differs by >250 SNPs to the reference genome, the assigned SNP address is 2.2.2.2.2.2.2 (no digits shared between both addresses, indicating that they differ by >250 SNPs).
- Samples ESC_LB5909AA_AS and ESC_LB5910AA_AS are inserted into the database. Both samples differ by >250 SNPs to the already inserted samples, but have no SNP differences compared to each other and are therefore both assigned to SNP address 3.3.3.3.3.3.3.
- Samples ESC_LB5918AA_AS (4.4.4.4.4.4.4), ESC_UB1905AA_AS (5.5.5.5.5.5.5), ESC_NB7682AA_AS (6.6.6.6.6.6.6), and ESC_LB2143AA_AS (7.7.7.7.7.7.7) are inserted into the database. Since all of these samples differ by >250 SNPs to all other samples, they are assigned to SNP addresses that do not share any digits with other samples in the database.
- Sample ESC_LB5911AA_AS (3.3.3.3.3.3.8) is inserted, which differs by 2 and 3 SNPs to samples ESC_LB5909AA_AS and ESC_LB5910AA_AS, respectively. This information is then represented in the SNP address for this sample, indicating that the number of SNP differences is between 1 and 5 since only the last digit is different (i.e., 3.3.3.3.3.3.3 and 3.3.3.3.3.3.8).
- In-house sequenced sample s_12480 (3.3.10.10.10.10.10) is introduced, which differs by ~70 SNPs to the already introduced samples starting with ‘3.3’, this is reflected in the SNP addresses which indicate differences between 51-100 SNPs (e.g., 3.3.10.10.10.10.10 and 3.3.3.3.3.3.3).
- In-house sequenced sample s_13150 (3.3.11.11.11.11.11) is introduced, which differs by ~70 SNPs to the already introduced samples starting with ‘3.3’, this is reflected in the SNP addresses which indicate differences between 51-100 SNPs (e.g., 3.3.11.11.11.11.11 and 3.3.3.3.3.3.3).
- Sample ESC_LB5912AA_AS (3.3.3.3.3.3.12) is introduced. This sample is the only tricky example since it differs by 5 SNPs to sample ESC_LB5909AA_AS (3.3.3.3.3.3.3) and ESC_LB5910AA_AS (3.3.3.3.3.3.3) and by 8 SNPs to sample ESC_LB5911AA_AS (3.3.3.3.3.3.8). As a consequence of the single linkage clustering algorithm (i.e., the sample is clustered based on the most closely related sample/cluster), the sample is assigned to a novel cluster with SNP address 3.3.3.3.3.3.12. These SNP addresses might be interpreted wrongly as ESC_LB5912AA_AS and ESC_LB5911AA_AS differing between 1-5 SNPs, while in reality, the SNP distance is 8. Because of the underlying principle of the single-linkage clustering algorithm, for a set of samples belonging to the same cluster within a given SNP threshold, a sample X will differ at most by that number of SNPs to at least one other sample Y of that cluster, while the same sample Y will differ at most by that number of SNPs to at least one other sample Z of that cluster. The total distance of X to Z can however then be more than that SNP threshold.

### **Figure S3: Alternative visualizations of the SNP addresses for cluster ST410.**

**Subfigure A**


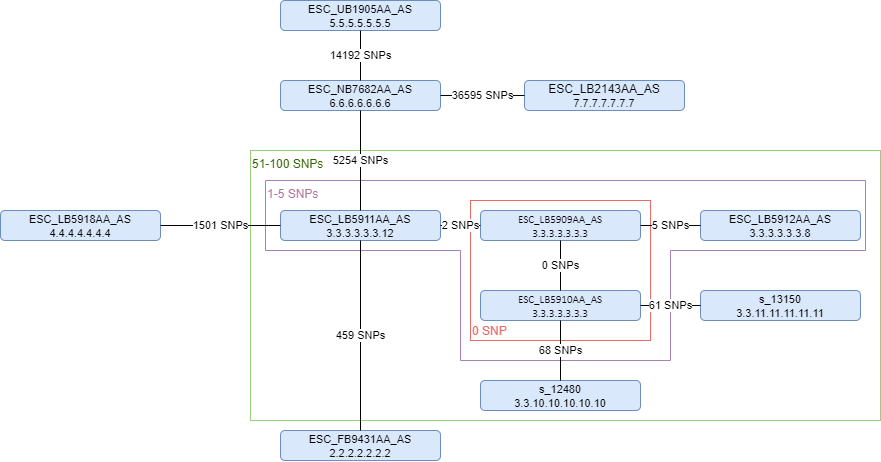


**Subfigure B**


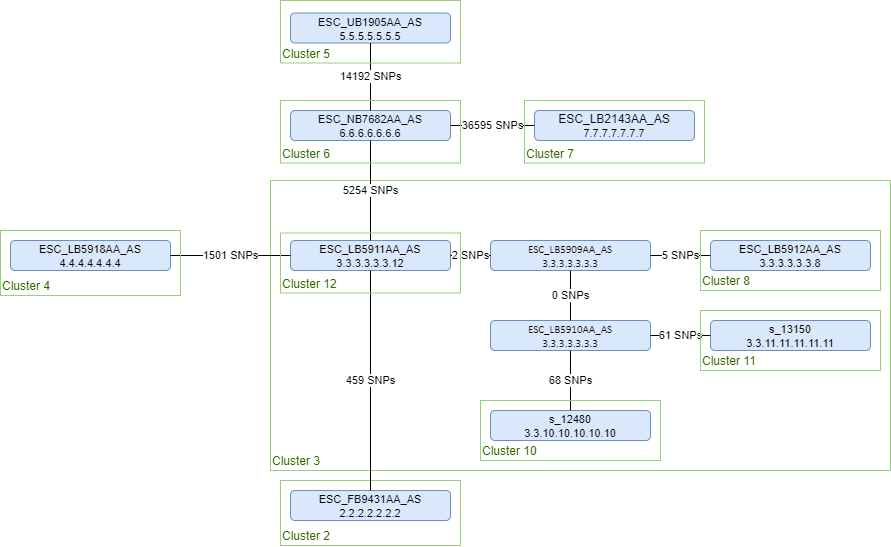


Each node represents a sample in the ST410 cluster. Nodes were connected to their closest neighbour based on the number of SNP differences (single-linkage clustering). In subfigure A, the colored rectangles represent the hierarchical grouping used to construct the SNP addresses. In subfigure B, each is individual cluster is indicated by a green rectangle.
